# The C-terminal HSP90 inhibitor NCT-58 kills trastuzumab-resistant breast cancer stem-like cells

**DOI:** 10.1038/s41420-021-00743-2

**Published:** 2021-11-13

**Authors:** Soeun Park, Yoon-Jae Kim, Jung Min Park, Minsu Park, Kee Dal Nam, Lee Farrand, Cong-Truong Nguyen, Minh Thanh La, Jihyae Ann, Jeewoo Lee, Ji Young Kim, Jae Hong Seo

**Affiliations:** 1 Division of Medical Oncology, Department of Internal Medicine, Korea University College of Medicine, Korea University, Seoul, 152-703 Republic of Korea; 2 Brain Korea 21 Program for Biomedical Science, Korea University College of Medicine, Korea University, Seoul, 152-703 Republic of Korea; 3 Department of Biomedical Research Center, Korea University Guro Hospital, Korea University, Seoul, 08308 Republic of Korea; 4 Adelaide Medical School, Faculty of Health and Medical Sciences, The University of Adelaide, Adelaide, SA 5000 Australia; 5 Laboratory of Medicinal Chemistry, College of Pharmacy, Seoul National University, Seoul, 08826 Republic of Korea

**Keywords:** Breast cancer, Apoptosis

## Abstract

N-terminal HSP90 inhibitors in development have had issues arising from heat shock response (HSR) induction and off-target effects. We sought to investigate the capacity of NCT-58, a rationally-synthesized C-terminal HSP90 inhibitor, to kill trastuzumab-resistant HER2-positive breast cancer stem-like cells. NCT-58 does not induce the HSR due to its targeting of the C-terminal region and elicits anti-tumor activity via the simultaneous downregulation of HER family members as well as inhibition of Akt phosphorylation. NCT-58 kills the rapidly proliferating bulk tumor cells as well as the breast cancer stem-like population, coinciding with significant reductions in stem/progenitor markers and pluripotent transcription factors. NCT-58 treatment suppressed growth and angiogenesis in a trastuzumab-resistant xenograft model, concomitant with downregulation of ICD-HER2 and HSF-1/HSP70/HSP90. These findings warrant further investigation of NCT-58 to address trastuzumab resistance in heterogeneous HER2-positive cancers.

## Introduction

Heat shock protein 90 (HSP90) is a chaperone that governs the maturation, stabilization, and activation of client proteins [1, 2]. HSP90 also interacts with a variety of pathways that can become oncogenic, and its aberrant activity is implicated in tumorigenesis and cancer progression [2–4]. For example, HSP90 has been shown to regulate the tyrosine kinase activity of human epidermal growth factor receptor 2 (HER2), facilitating pro-survival signaling [5–7].

Trastuzumab is a humanized anti-HER2 monoclonal antibody used for the treatment of HER2-positive breast cancer, however, most patients eventually develop resistance to the drug within 1–2 years [8–10]. Key mechanisms responsible for trastuzumab resistance include HER2/HER3 and HER2/EGFR interactions, hyperactivation of PI3K/Akt signaling, and accumulation of truncated forms of HER2 [10, 11]. Truncated p95HER2 is a constitutively active form of the tyrosine kinase that activates downstream signaling through dimerization with other HER family members [11–13]. These trastuzumab resistance-related factors are HSP90 client proteins [5–7, 14], and therefore the inhibition of HSP90 may suppress several potent oncogenic drivers and trastuzumab-refractory factors.

HER2-positive tumors are typically highly heterogeneous consisting of differentiated tumor bulk cells and a smaller subset of breast cancer stem cells (BCSCs) with tumorigenic potential and asymmetric cell division capability [15, 16]. A positive association has been reported between CSCs and trastuzumab resistance in HER2-positive cancer [17–19]. Expression of the stem/progenitor cell marker ALDH1 is highly elevated in HER2-positive breast cancer and is associated with an aggressive phenotype [20, 21]. Subpopulations with CD44^high^/CD24^low^ mesenchymal stem-like phenotypes are often resistant to trastuzumab [18, 22, 23]. Therefore, new therapeutic strategies that effectively target both cancer stem cells and trastuzumab resistance are needed to improve clinical outcomes. In addition, recent studies have shown that HSP90 confers stability to the pluripotent transcription factors Oct4 and Nanog by preventing degradation via the ubiquitin-proteasome pathway, further highlighting the potential advantages of HSP90 inhibition with regard to attenuating pluripotency and self-renewal capacity [24, 25].

Although HSP90 is a promising target for cancer treatment, there are no approved inhibitors due to issues including heat shock response (HSR) induction and off-target effects [26–28]. Inhibition of HSP90 by binding to its N-terminal domain also triggers heat shock factor-1 (HSF-1)-mediated HSP transcription, leading to upregulation of HSP27, HSP40, HSP70, and HSP90 [2, 3, 27]. Collectively, this series of reactions is called the HSR and is an intracellular defense mechanism that promotes survival of malignant cells.

**Fig. 1:**
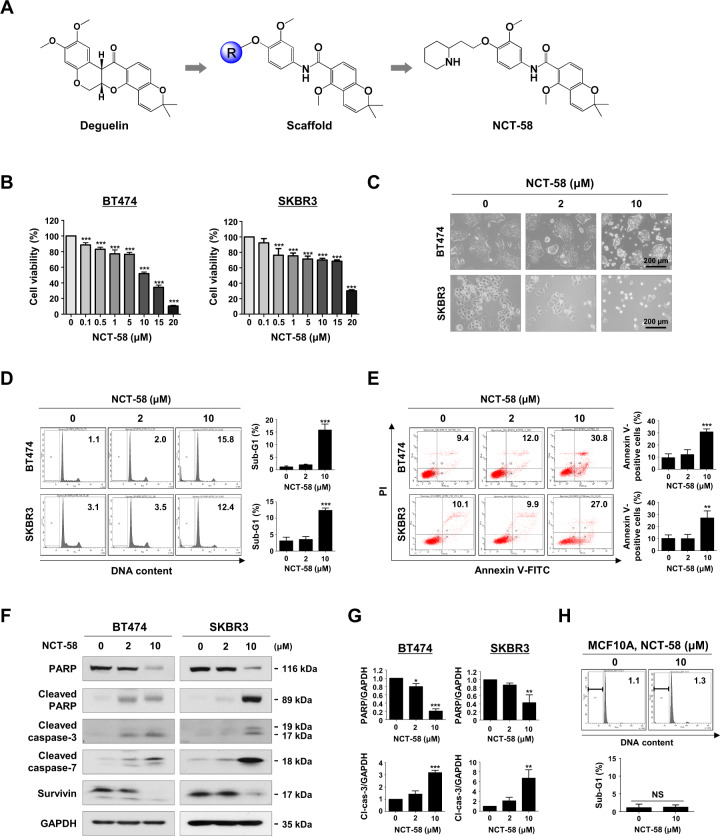
NCT-58 reduces cell viability and induces apoptosis in HER2-positive breast cancer cells. **A** Chemical structure of deguelin and NCT-58. **B** BT474 and SKBR3 cells were treated with various concentrations of NCT-58 (0.1–20 μM) or DMSO (solvent control) for 72 h. Cell viability was determined by MTS assay (***p < 0.001, *n* = 4). **C** Morphological changes of BT474 and SKBR3 cells after treatment with NCT-58 (2–10 μM, for 72 h) as seen through phase-contrast microscopy. **D** Cells were treated with NCT-58 (2–10 μM) for 72 h and the sub-G1 population was assessed by flow cytometry (***p < 0.01, *n* = 3). **E** Early and late apoptotic cells in the presence or absence of NCT-58 were quantified by annexin V/PI staining (right panel, **p < 0.01; ***p < 0.001, *n* = 3). **F** Effect of NCT-58 on expression of cleaved-caspase-3, cleaved-caspase-7, cleaved-PARP and survivin in BT474 and SKBR3 cells. **G** Quantitative graphs of these protein levels (*p < 0.05; **p < 0.01; ***p < 0.001, *n* = 3). GAPDH was used as an internal loading control. **H** The sub-G1 fraction of the normal human mammary epithelial MCF10A cells was analyzed through flow cytometry after exposure to 10 μM of NCT-58 for 72 h (NS, not significant, versus DMSO control, *n* = 3). The results are presented as mean ± SD of at least three independent experiments and analyzed by one-way ANOVA or Student’s *t*-test followed by Bonferroni’s post hoc test.

We developed the novel C-terminal HSP90 inhibitor NCT-58 to address the shortcomings of N-terminal HSP90 inhibitors. NCT-58 (compound 80) is one of 90 synthesized *O*-substituted analogues of the B- and C-ring truncated scaffold of deguelin, which is a naturally occurring C-terminal inhibitor (Fig. 1A). NCT-58 specifically binds to the C-terminal domain of HSP90 and was identified through a molecular docking study [29]. We sought to further evaluate its efficacy against cancer stem cells in trastuzumab-resistant HER2-positive breast cancer.

## Results

### NCT-58-dependent apoptosis is mediated by caspase activation in HER2-positive breast cancer cells

We first sought to evaluate the effect of NCT-58 on cell viability and apoptosis in HER2-positive breast cancer cells. NCT-58 treatment (0.1–20 μM for 72 h) dose-dependently reduced cell viability in HER2-positive BT474 and SKBR3 cells (Fig. 1B). NCT-58 (2–10 μM, 72 h) elicited morphological features of apoptosis (Fig. 1C), including a marked accumulation of cells in the sub-G1 phase (Fig. 1D) and an increase in the number of early and late apoptotic cells (Fig. 1E). These effects were accompanied by caspase-3/−7 activation and PARP cleavage, as well as downregulation of survivin (Fig. 1F, G, and Supplementary Fig. S1). NCT-58 did not affect normal human mammary epithelial MCF10A cells, as determined by sub-G1 cell cycle analysis (Fig. 1H).

### NCT-58 targets the C-terminal domain of HSP90 and downregulates the expression of EGFR/HER2/HER3

**Fig. 2:**
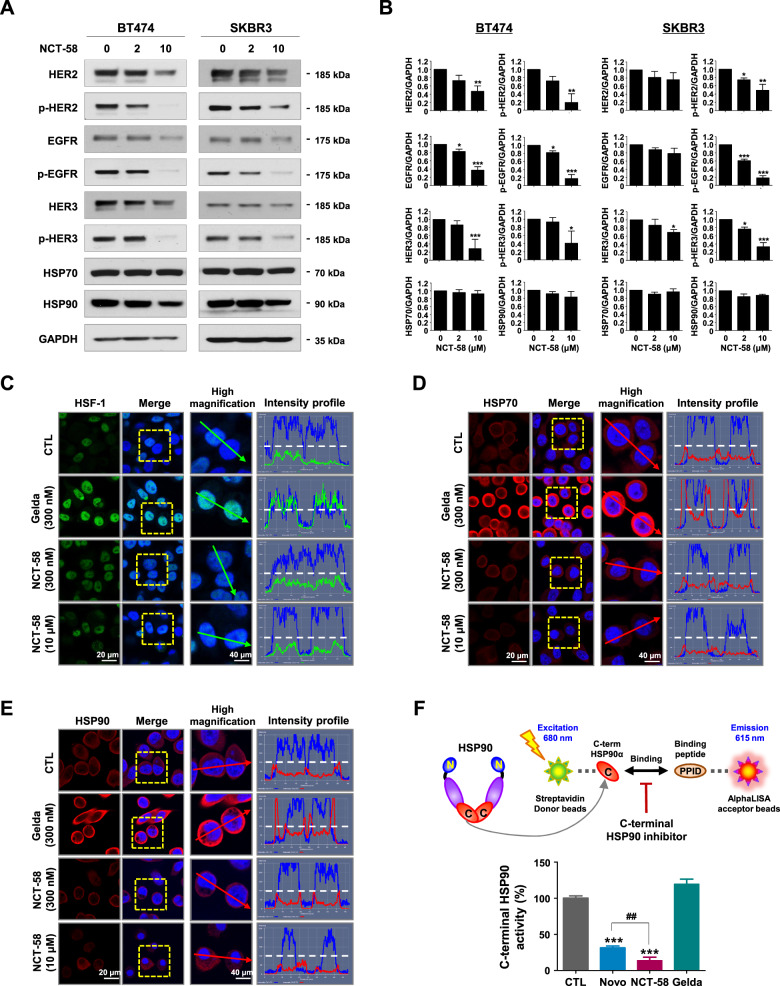
NCT-58 targets the C-terminal domain of HSP90 and downregulates HER2, HER3, and EGFR expression. **A** Immunoblot analyses of HER2, phospho-HER2 (Tyr1221/1222), EGFR, phospho-EGFR (Tyr1068), HER3, phospho-HER3 (Tyr1289), HSP70, and HSP90 protein expression in BT474 and SKBR3 cells following exposure to NCT-58 (0–10 μM, 72 h). GAPDH was used as a loading control. **B** Quantitative graphs represent the ratio of expression of HER2 family members, HSP70 and HSP90 relative to GAPDH expression in the presence or absence of NCT-58 (*p < 0.05; **p < 0.01; ***p < 0.001, *n* = 3). **C**–**E** Com*p*arison of the effects of NCT-58 and geldanamycin on induction of HSR. SKBR3 cells were treated with NCT-58 (300 nM and 10 µM) or geldanamycin (300 nM) for 24 h. Cells were immunostained for HSF-1 (green, **C**), HSP70 (red, **D**) or HSP90 (red, **E**) with DAPI (nuclei, blue). Fluorescence intensity of these proteins is represented in arbitrary units as defined by the software using the intensity profile tool. **F** Potency of C-terminal HSP90 inhibition (novobiocin, geldanamycin, and NCT-58) was determined with an HSP90α (C-terminal) inhibitor screening assay. The inhibitory effect of each drug (500 μM) on recombinant HSP90α (C-terminal):PPID binding activity was measured with an AlphaScreen microplate assay (***p < 0.001, CTL vs NCT-58 or Novo; ^##^*p* < 0.01, Novo vs NCT-58, *n* = 3). CTL control, Novo novobiocin, Gelda geldanamycin, PPID peptidylprolyl isomerase D.

We examined whether NCT-58 regulates HSP90 major client proteins including HER family members. The expression and phosphorylation levels of HER2, EGFR, and HER3 were determined by immunoblot analysis. NCT-58 treatment was found to downregulate the expression levels and phosphorylation of HER2 (Tyr1221/1222), HER3 (Tyr1289), and EGFR (Tyr1068) in BT474 and SKBR3 cells (Fig. 2A, B). Immunoblot analyses also showed that NCT-58 (2–10 μM, 72 h) did not impact HSP70 and HSP90 expression in HER2-positive breast cancer cells (Fig. 2A, B).

To investigate whether NCT-58 induces the HSR, the subcellular localization of HSF-1 and the expression levels of HSP70 and HSP90 following NCT-58 treatment were determined by immunocytochemical analysis. SKBR3 cells were treated with geldanamycin (300 nM), a known N-terminal HSP90 inhibitor, and NCT-58 (300 nM and 10 μM) for 24 h and immunostained for HSF-1, HSP70, and HSP90. No increase in HSF-1 was observed following exposure to NCT-58, whereas a marked increase in nuclear accumulation of HSF-1 occurred after geldanamycin treatment (Fig. 2C). NCT-58 did not affect HSP70 or HSP90, while geldanamycin markedly upregulated HSP70 (Fig. 2D) and HSP90 (Fig. 2E). We further confirmed that NCT-58 had no effect on HSP70 and HSP90 expression in HER2-positive breast cancer cells at 24 h, as determined by immunoblotting (Supplementary Fig. S2). NCT-58 competitively inhibited binding between HSP90α CTD and its co-chaperone peptidylprolyl isomerase D (PPID) to a greater extent than geldanamycin and novobiocin, a well-characterized C-terminal HSP90 inhibitor (Fig. 2F).

### NCT-58 kills trastuzumab-resistant cells and suppresses HER family members

**Fig. 3:**
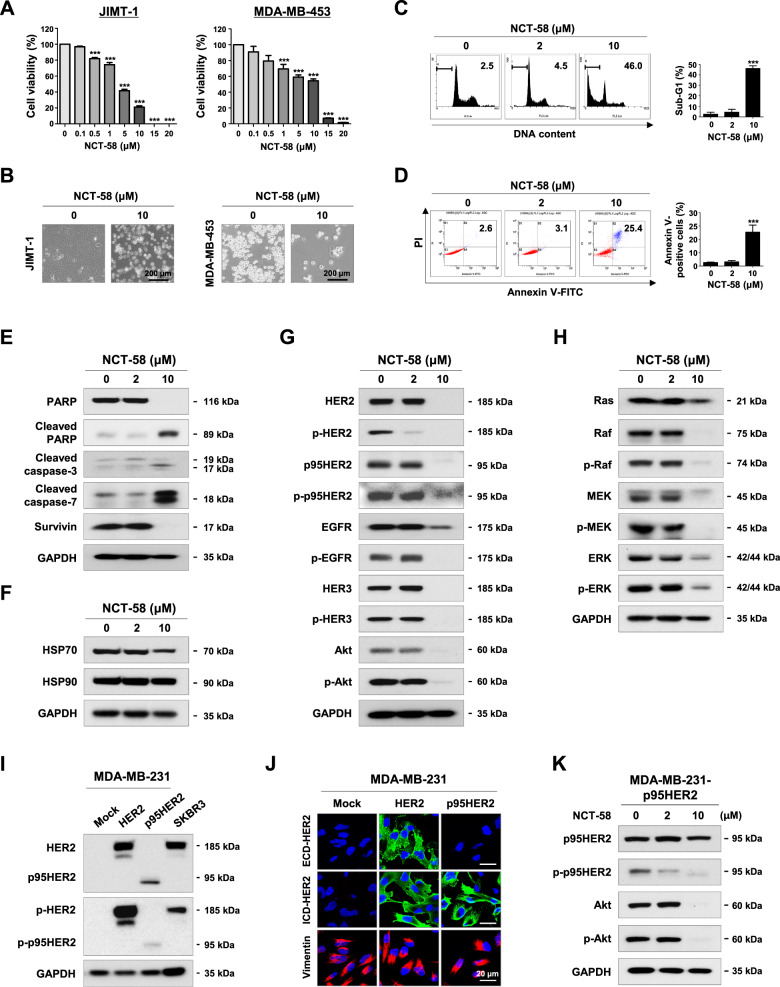
NCT-58 induces apoptosis and inhibits tyrosine kinase activity of HER2 in trastuzumab-resistant cells. **A** NCT-58 (0.1–20 μM) reduced cell viability in trastuzumab-resistant JIMT-1 and MDA-MB-453 cells (***p < 0.001, *n* = 4). **B** Morphological changes in these cells as seen through phase-contrast microscopy in the presence or absence of NCT-58 (10 μM, 72 h). **C**, **D** NCT-58 treatment (2–10 μM, 72 h) resulted in a significant induction of apoptosis in JIMT-1 cells, as evidenced by increased sub-G1 accumulation (**C**, ***p < 0.001, *n* = 3) and annexin V/PI-positive cells (**D**, ***p < 0.001, *n* = 3). **E** Effects of NCT-58 (2–10 μM, 72 h) on expression of PARP, cleaved-PARP, cleaved-caspase-3, cleaved-caspase-7, and survivin in JIMT-1 cells. **F** Influence of NCT-58 (2–10 μM, 72 h) on protein contents of HSP70 and HSP90 in JIMT-1 cells. **G** Immunoblot analyses of HER2, phospho-HER2, p95HER2, phospho-p95HER2, EGFR, phospho-EGFR, HER3, phospho-HER3, Akt and phospho-Akt (Ser473) expression in JIMT-1 cells following exposure to NCT-58 (2–10 μM, 72 h). **H** Effects of NCT-58 (2–10 μM, 72 h) on Ras, Raf, phospho-Raf, Mek, phospho-Mek, Erk, and phospho-Erk protein expression in JIMT-1 cells. **I** Immunoblot analyses of HER2, p95HER, phospho-HER2, and phospho-p95HER2 in both HER2- and p95HER2-overexpressing MDA-MB-231 cells. **J** Immunofluorescence analysis of HER2 and p95HER2. Cells were immunostained with ECD-HER2 (green) or ICD-HER2 (green, CB11) antibody and counterstained with DAPI (blue). Vimentin (red) was stained to demonstrate cellular features in MDA-MB-231 cells. **K** The levels of p95HER2, phospho-p95HER2, Akt, and phospho-Akt were downregulated in p95HER2-overexpressing MDA-MB-231 cells after exposure to NCT-58 (2–10 μM, 72 h).

We further evaluated the impact of NCT-58 on trastuzumab resistance using JIMT-1 and MDA-MB-453 cells, which are trastuzumab-resistant cell lines. NCT-58 treatment (0.1–20 μM, 72 h) significantly suppressed the viability of JIMT-1 and MDA-MB-453 cells in a dose-dependent manner (Fig. 3A). NCT-58 (10 μM) elicited apoptotic morphological changes with cytosolic shrinkage (Fig. 3B), while flow cytometry analysis revealed that NCT-58 caused a marked increase in the sub-G1 population (Fig. 3C) and early/late apoptosis (Fig. 3D) in JIMT-1 cells. These responses were associated with increased caspase-3/-7 activation and downregulation of PARP and survivin in JIMT-1 cells (Fig. 3E and Supplementary Fig. S3). Following exposure to NCT-58, the expression and phosphorylation of HER2/HER3/EGFR were significantly reduced in trastuzumab-resistant JIMT-1 cells (Fig. 3G and Supplementary Fig. S4), but without upregulation of HSP70 and HSP90 (Fig. 3F and Supplementary Fig. S5).

The Ras/Raf/mitogen-activated protein kinase pathway (MAPK) signaling cascade is activated by dimerization of HER family members and plays a crucial role in various aspects of breast cancer progression including the cell cycle, proliferation, apoptosis, and angiogenesis [11, 30]. Furthermore, Ras, Raf, Mek, and Erk are client proteins of HSP90, and their expression and activation are also controlled by HSP90 [31]. We observed that NCT-58 significantly impaired their expression as well as the phosphorylation of Ras, Raf (Ser338), Mek (Ser217/221) and Erk (Tyr202/204) in JIMT-1 cells (Fig. 3H and Supplementary Fig. S6).

It is noteworthy that NCT-58 exposure effectively reduced the levels of truncated p95HER2 and its phosphorylated form, as well as downregulation of Akt and phospho-Akt (Ser473) protein contents in JIMT-1 (Fig. 3G and Supplementary Fig. S4). We further confirmed this phenomenon in p95HER2-overexpressing MDA-MB-231, a typical triple-negative breast cancer (TNBC) cell line (Fig. 3I). HER2-overexpressing cells expressed both ECD- and ICD-HER2, whereas p95HER2-overexpressing cells specifically expressed ICD-HER2 (Fig. 3J). Following NCT-58 treatment, p95HER2 expression and phosphorylation was dramatically reduced in the p95HER2-overexpressing MDA-MB-231 cells, and levels of Akt and p-Akt were also significantly reduced (Fig. 3K and Supplementary Fig. S7).

### NCT-58 eradicates HER2-positive BCSCs without triggering the HSR

**Fig. 4:**
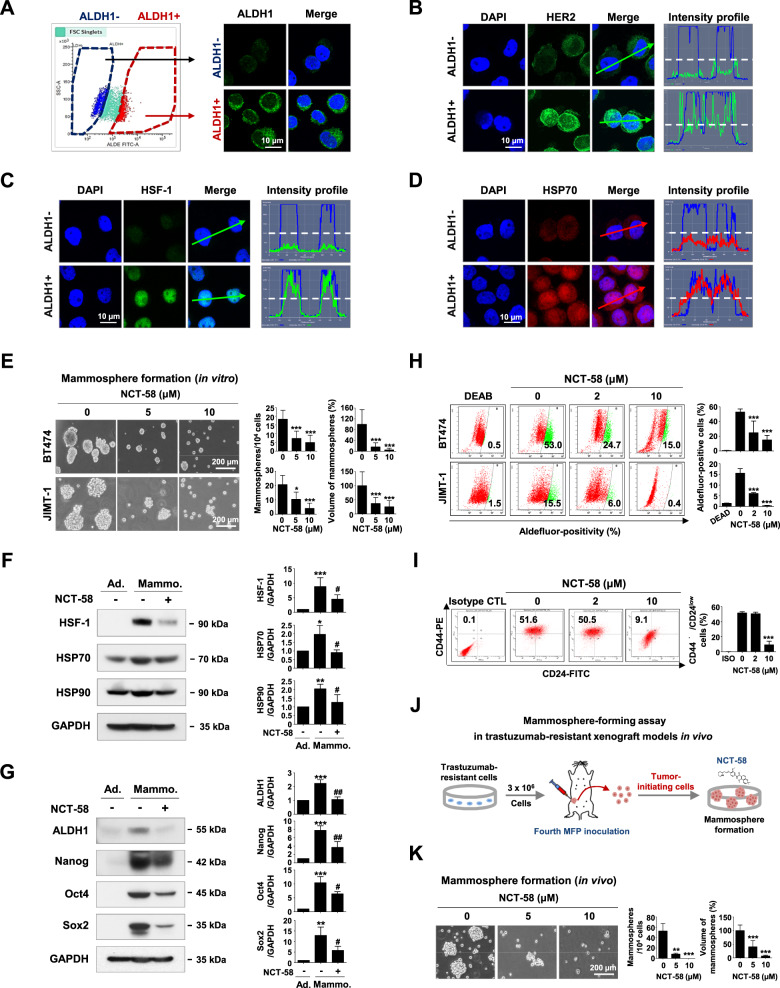
NCT-58 targets HER2-positive BCSC-like properties. **A**–**D** ALDH1 low (ALDH1-) and high (ALDH1+) populations were sorted from BT474 cells using FACS. Cells were immunostained for ALDH1 (green, **A**), HER2 (green, **B**), HSF-1 (green, **C**) and HSP70 (red, **D**) with DAPI (blue). Intensity was analyzed by confocal microscopy using the intensity profiling tool. The straight line (white dotted line) indicates 100 intensity units (y-axis on the left, a range scale 0–260 unit). **E** Effect of NCT-58 on mammosphere formation by BT474 and JIMT-1 cells. Cells (5 × 10^4^ cells/mL) were cultured in ultralow attachment plates in the presence or absence of NCT-58 (5 and 10 μM) for 3 and 7 days, respectively. The number and volume of mammospheres was quantified by optical microscopy (*p < 0.05; ***p < 0.001, *n* = 3). **F**–**G** BT474 cells were cultured in normal culture medium or serum-free suspension conditions in the presence or absence of NCT-58 (10 μM) for 3 days. **F** Changes in HSF-1, HSP70, and HSP90 levels as determined by immunoblotting. Quantitation of these protein levels is shown in the right panels [*p < 0.05; **p < 0.01; ***p < 0.001, adherent cells (Ad.) vs mammospheres (Mammo.); ^#^*p* < 0.05, DMSO control vs NCT-58 treatment in mammospheres, *n* = 3]. **G** Expression levels of ALDH1, Nanog, Oct4, and Sox2 as detected by immunoblotting. Quantitation of the expression of these proteins is shown [***p < 0.001, Ad. vs Mammo.; ^#^*p* < 0.05; ^##^*p* < 0.01, DMSO control vs NCT-58 treatment in mammospheres, *n* = 3]. GAPDH was used as an internal loading control. **H**, **I** Influence of NCT-58 (2–10 µM, for 72 h) on ALDH1 activity in BT474 and JIMT-1 cells and CD44^high^/CD24^low^ stem-like phenotypes in JIMT-1 cells. Quantitative graph of percentages of Aldefluor-positivity (**H**, ***p < 0.001, *n* = 3) or CD44^high^/CD24^low^ populations (**I**, ***p < 0.001, *n* = 3) are shown, respectively. **J**, **K** Im*p*act of NCT-58 on mammosphere formation in a JIMT-1 xenograft model. Dissociated single cells (5 × 10^4^ cells/mL) from xenograft tumors (200–250 mm^3^) were plated in ultralow attachment dishes and cultured in the presence or absence of NCT-58 (5 and 10 μM) for 8 days. The number and volume of mammospheres was quantified (**p < 0.01; ***p < 0.001, *n* = 3). The results are presented as mean ± SD of at least three independent experiments and analyzed by one-way ANOVA followed by Bonferroni’s post hoc test.

HSF-1 plays a major role in the maintenance of BCSC-like properties [32]. ALDH1-positive or –negative cells were sorted from HER2-positive BT474 and JIMT-1 cell populations, respectively, and the levels of HSR-related factors were examined (Fig. 4A and Supplementary Fig. S8A). In agreement with previous findings [33], HER2 was preferentially overexpressed in the ALDH1-positive population (Fig. 4B and Supplementary Fig. S8B). Immunocytochemistry analysis and intensity profiling revealed that HSF-1 is highly elevated and accumulates in the nucleus of ALDH1-positive cells, whereas ALDH1-negative cells exhibit comparatively lower levels of HSF-1 (Fig. 4C and Supplementary Fig. S8C). Considerable overexpression of HSP70 was found in ALDH1-positive cells, implying that abundant HSF-1 likely enhances the transcription of HSP70 in the BCSC subpopulation (Fig. 4D and Supplementary Fig. S8D).

Mammospheres are enriched in mammary stem/progenitor populations that possess self-renewal and differentiation potential, as well as higher ALDH1 activity [21, 34]. The mammosphere-forming ability of BT474 and JIMT-1 was diminished in the presence of NCT-58 (Fig. 4E). It is noteworthy that HSF-1 protein content was significantly upregulated in BCSC-enriched mammospheres and this effect was considerably downregulated by NCT-58 treatment. NCT-58 also significantly downregulated HSP70 and HSP90 protein content (Fig. 4F). Levels of the pluripotent transcription factors Nanog, Oct4, and Sox2 as well as ALDH1 were markedly diminished in the presence of NCT-58 (Fig. 4G).

After exposure to NCT-58, a dose-dependent reduction in Aldefluor-positive cells was observed in both trastuzumab-sensitive BT474 and -resistant JIMT-1 cells (Fig. 4H), together with marked reductions in the JIMT-1 CD44^high^/CD24^low^ population (Fig. 4I). It is noteworthy that formation of mammospheres derived from trastuzumab-resistant xenograft tumor was significantly suppressed by NCT-58 treatment (Fig. 4J, K)

### NCT-58 administration suppresses trastuzumab-resistant tumor growth

**Fig. 5:**
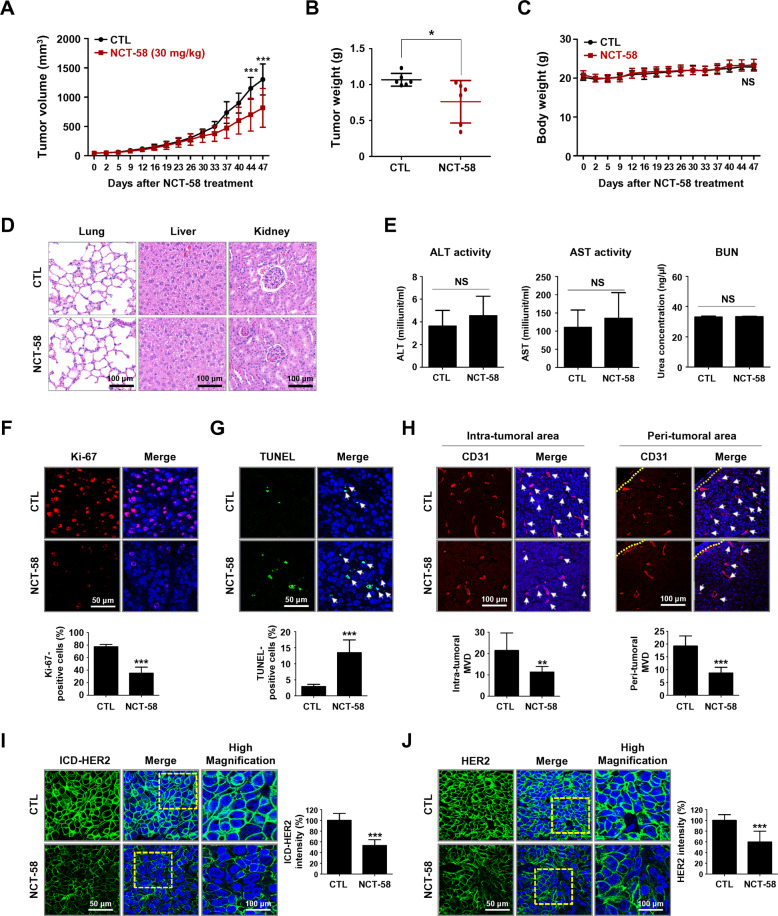
NCT-58 inhibits tumor growth in trastuzumab-resistant JIMT-1 xenografts. **A**–**C** Effect of NCT-58 on tumor growth in vivo. JIMT-1 cells (3 × 10^6^) were injected into the mammary fat pads of BALB/c nude mice. Mice were administered intraperitoneally with NCT-58 (30 mg/kg·BW, every other day) or solvent control for 47 days (*n* = 6/each group). Tumor volumes were measured with a caliper at the intervals indicated. **A**, **B** NCT-58 administration resulted in significant decreases in tumor growth (**A**, ***p < 0.001, *n* = 6) and tumor weight (**B**, *p < 0.05, *n* = 6). **C** Changes in body weight of the xenografted mice after exposure to NCT-58 or control vehicle (NS; not significant, *n* = 6). **D** Representative histological analysis of lung, liver, and kidney sections stained with hematoxylin and eosin (H&E) and analyzed by microscope slide scanner. **E** Effects of NCT-58 on serum biochemical parameters of liver and kidney function. Blood biochemical analysis indicated there was no significant change in serum ALT, AST, BUN (NS; not significant). ALT, alanine aminotransferase; AST, aspartate aminotransferase; BUN, blood urea nitrogen. **F** Influence of NCT-58 on Ki-67 expression. Tumor tissue sections were immunostained for Ki-67 (red) and DAPI (blue). The graph shows the percentage of Ki-67-positive cells (***p < 0.001, *n* = 6). **G** NCT-58-induced apoptosis was determined by TUNEL assay. Extent of apoptosis expressed as the percentage of total TUNEL-positive cells (***p < 0.001, *n* = 6). **H** Effect of NCT-58 on tumor angiogenesis, as determined by a microvessel density (MVD) assay. Tumor tissues were immunostained with a specific endothelial marker CD31 (red) and DAPI (blue). The number of CD31-positive microvessels in the intratumoral and peritumoral areas were quantified, respectively (**p < 0.01; ***p < 0.001, *n* = 6). **I**, **J** Immunohistochemical analysis for the intracellular domain (ICD)-HER2 (green, **I**) and full-length HER2 (green, **J**) in vivo. Quantitative graphs of signal intensities shown in the right panels (***p < 0.001, *n* = 6). The results are presented as mean ± SD and data were analyzed by unpaired Student’s *t*-test and two-way ANOVA followed by Bonferroni’s post hoc test.

To confirm the physiological relevance of our in vitro observations, we evaluated the impact of NCT-58 on tumor angiogenesis, expression of BCSC makers and tumor growth in a trastuzumab-resistant xenograft model. JIMT-1 cells (3 × 10^6^) were orthotopically injected into the right fourth mammary fat pads of BALB/c female nude mice (*n* = 6, each group). The mice were treated with NCT-58 (30 mg/kg body weight, every other day) or vehicle control (1:9, DMSO:corn oil). NCT-58 administration caused a significant impediment of tumor growth (Fig. 5A) and a marked decrease in tumor weight (Fig. 5B). There were no significant differences in body weight between the groups (Fig. 5C). No histological abnormalities were observed in the lungs, liver, and kidneys after administration of NCT-58 (Fig. 5D). To examine the potential organ toxicity of NCT-58, aspartate aminotransferase (AST), alanine aminotransferase (ALT) and blood urea nitrogen (BUN) assays were performed with serum samples from the animals. No significant changes were found between the control and treatment groups, suggesting that the inhibitor does not overtly affect liver or kidney function (Fig. 5E).

The antitumor effect of NCT-58 was observed concurrently with a marked reduction in Ki-67-positive cells (Fig. 5F) and a significant increase in apoptosis as detected by TUNEL-positivity (Fig. 5G). To further evaluate the effect of NCT-58 on tumor angiogenesis, a microvessel density (MVD) assay was performed using the endothelial-specific marker CD31 [35]. NCT-58 administration significantly reduced the number of CD31-positive vessels in both peritumoral and intratumoral areas (Fig. 5H). Consistent with in vitro observations, NCT-58 administration significantly suppressed the expression of both ICD-HER2 (Fig. 5I) and full-length HER2 (Fig. 5J).

### Anti-tumor effect of NCT-58 is accompanied by the suppression of BCSC-like characteristics and downregulation of HSF-1/HSP70/HSP90

**Fig. 6:**
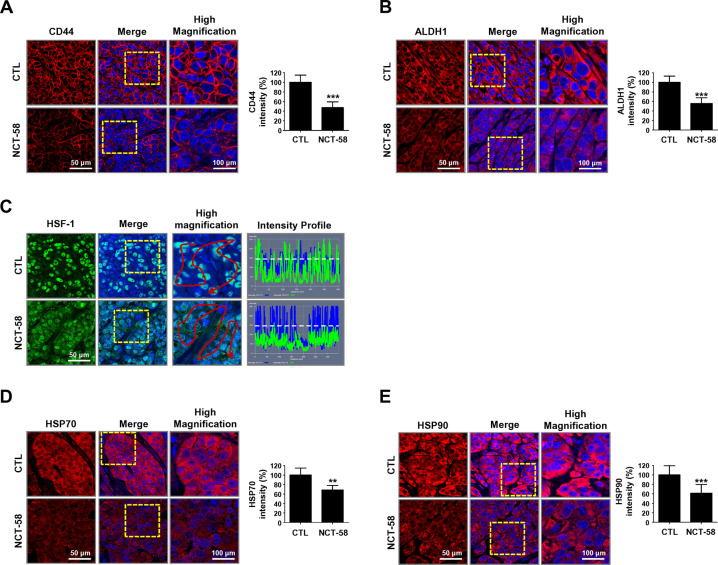
NCT-58 suppresses BCSC-like properties and downregulates the expression of HSF-1, HSP70, and HSP90 in vivo. **A, B** Following exposure to NCT-58, CD44 and ALDH1 expression levels were markedly downregulated in JIMT-1 xenograft tumors. The fluorescence intensities of CD44 (**A**, ***p < 0.001, *n* = 6) and ALDH1 (**B**, ***p < 0.001, *n* = 6) were analyzed using a histogram tool. **C**–**E** Effect of NCT-58 on induction of the HSR in vivo. **C** NCT-58 administration resulted in a decrease in nuclear expression of HSF-1 (green). Fluorescence intensity of HSF-1 localized in the nuclei is represented in arbitrary units as defined by the software using the intensity profile tool. **D**, **E** NCT-58 downregulated both HSP70 and HSP90 expression. Quantitative graphs of fluorescence intensities of HSP70 (**D**, **p < 0.01, *n* = 6) and HSP90 (**E**, ***p < 0.001, *n* = 6) signal, shown in the bottom panels, respectively. Tumors were harvested within 24 h of the last administration of NCT-58 for immunohistochemistry assessment. Data were analyzed by unpaired Student’s *t*-test.

The cell surface glycoprotein CD44, as a marker of trastuzumab resistance [11, 18], is highly expressed in the plasma membrane of JIMT-1 control tumor cells. Animals receiving NCT-58 exhibited a noticeably lower level of CD44 in vivo (Fig. 6A). Furthermore, ALDH1 expression was also significantly different between the groups (Fig. 6B).

HSF-1 activity was suppressed after NCT-58 treatment, as evidenced by a significant decrease in the signal intensity of nuclear HSF-1 (Fig. 6C) as well as downregulation of its downstream client proteins HSP70 (Fig. 6D) and HSP90 (Fig. 6E).

## Discussion

HSP90 orchestrates the activation and stability of more than 200 potential client oncoproteins [1]. However, N-terminal inhibitors of this target have been shown to induce the HSR, representing a considerable obstacle in clinical development [26, 27].

The master transcriptional determinant HSF-1 plays a pleiotropic role not only in regulating the HSR, but also during tumorigenesis, tumor cell migration, and metastasis [36–38]. Clinical evidence has shown significant associations between HSF-1 and HER2 in HER2-positive breast cancer, while higher levels of nuclear HSF-1 are correlated with histologic grade, larger tumor sizes and reduced survival [39]. HSF-1 in HER2/Neu+ transgenic mice also stimulates tumorigenesis in the mammary glands, and metastasis via the promotion of epithelial to mesenchymal transition (EMT) [38]. Activated HSF-1 translocates to the nucleus to promote the transcription of HSPs during oncogenesis, facilitating rapid tumor cell proliferation [40]. Meanwhile, HSP70 hinders the apoptosis pathway by interfering with the release of cytochrome c, inhibiting apoptosome complex formation and caspase activation [41].

Treatment with NCT-58 did not induce the HSR, as evidenced by the absence of nuclear accumulation of HSF-1 and upregulation of HSP70 expression in HER2-positive breast cancer cells. NCT-58 significantly increased apoptosis via caspase-3/caspase-7 activation, but elicited no such toxicity in non-malignant cells. Our in vivo findings show that NCT-58 administration suppresses tumor growth, concomitant with increased apoptosis and simultaneous downregulation of HSF-1/HSP70/HSP90. Meanwhile, no histological changes were seen in liver and kidney tissue sections.

Evidence suggests that trastuzumab resistance can be attributable to the existence of a subpopulation of BCSCs in HER2-positive breast cancers [42, 43]. The CD44/hyaluronan complex masks the cognate epitope, interfering with trastuzumab binding to HER2, and leading to PI3K/Akt activation and trastuzumab resistance [11, 18]. We have previously found that trastuzumab-sensitive cell lines harbor limited numbers of CD44^high^/CD24^low^ cells at less than 1% [44], whereas trastuzumab-resistant JIMT-1 cells harbor considerably higher numbers at more than 50%, suggesting that the CD44^high^/CD24^low^ phenotype is a marker of trastuzumab resistance. Trastuzumab-resistant JIMT-1 cells when treated with NCT-58 exhibited a noticeable reduction in the CD44^high^/CD24^low^ population and ALDH1 activity, as well as suppression of mammosphere formation. HSP90 physically interacts with Nanog and Oct4, protecting them from ubiquitin-mediated proteasomal degradation, implying that HSP90 may contribute to BCSC self-renewal [24, 45, 46]. Elevated levels of Nanog, Oct4, and SOX2 in BCSC-enriched mammospheres were overwhelmingly abolished after NCT-58 challenge.

Emerging evidence suggests that HSF-1 plays a pivotal role in regulating the BCSC phenotype [32, 45]. The BCSC-enriched population exhibited relatively higher levels of HSF-1 in both mammospheres and ADLH1-positive cells. Highly abundant HSF-1 is believed to enhance the expression of HSPs such as HSP70 and HSP90 via HSF-1-mediated transcription, which can promote BCSC survival. In agreement with our observations, forced overexpression of HSF-1 increased mammosphere-forming ability as well as CD44, Sox2 and ALDH1 expression, and conferred drug resistance in breast cancer, while HSF-1 knockdown attenuated these phenomena [32, 47]. Further defining the role of HSF-1 in regulating the BCSC phenotype will likely provide important clues into developing effective CSC-targeted therapeutic strategies.

The emergence of acquired trastuzumab resistance remains an urgent unmet medical need. Oncogenic p95HER2 retains tyrosine kinase activity and interacts with HER3, enhancing activation of Akt signaling and tumor cell survival [11, 12, 48]. Akt directly interacts with and phosphorylates HSF-1 at S326, which confers HSF-1 activation, leading to EMT via Slug upregulation [38, 49, 50]. Importantly, NCT-58 downregulates the levels of truncated p95HER2 and Akt levels and phosphorylation in trastuzumab-resistant HER2-positive cells, an effect also seen in p95HER2-overexpressing MDA-MB-231 cells. It is conceivable that Akt downregulation by NCT-58 may attenuate the transcriptional ability of HSF-1, resulting in downregulation of HSP70/HSP90.

**Fig. 7:**
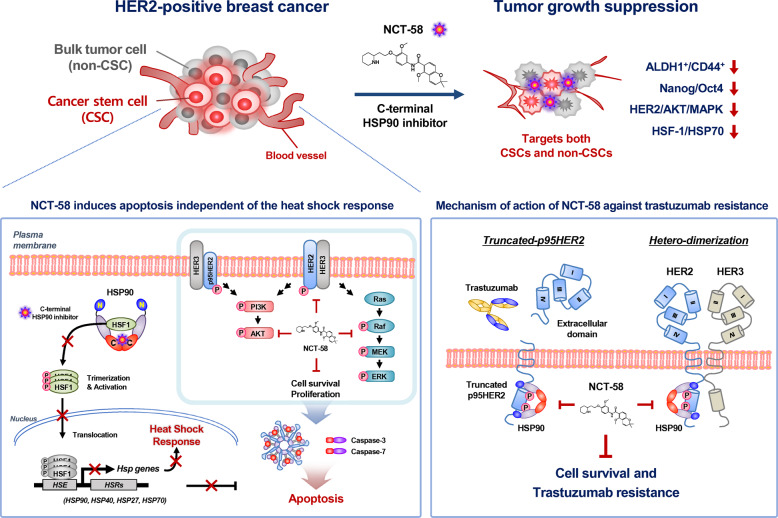
Hypothetical model illustrating multiple actions of NCT-58 on cancer stem-like properties, HER2 signaling, and trastuzumab resistance in HER2-positive breast cancer. **i** NCT-58 targets the C-terminal domain of HSP90 independent of the HSR and effectively induces apoptosis via the activation of cleaved caspase-3/7. **ii** NCT-58 downregulates the expression and phosphorylation of HER2, HER3, and EGFR as well as suppresses truncated p95HER2 accumulation and Akt activation, concomitant with downregulation of the Ras/Raf/Mek/Erk signaling pathway. **iii** NCT-58 kills not only proliferating tumor cells, but also eliminates BCSC-like cells. The latter responses are accompanied by the reduction of stem/progenitor markers CD44/ALDH1 and expression of the pluripotent transcription factors Nanog/Oct4/Sox2 as well as downregulation of HSF-1/HSF70/HSP90. [Heat shock response, HSR; Heat shock factor-1, HSF-1, Heat shock element, HSE].

In conclusion, NCT-58 does not induce the HSR as targeting of the C-terminal region is not accompanied by HSF-1-mediated HSP70 and HSP90 upregulation that occurs when targeting the N-terminus [27]. NCT-58 suppresses HER2-positive breast cancer cells with simultaneous inhibition of important trastuzumab resistance factors including HER2/HER3/Akt and truncated p95HER2 as well as downregulation of Ras/Raf/Mek/Erk. In Fig. 7, we present a hypothetical model illustrating several key actions of NCT-58 on cancer stem-like properties, HER2 signaling, and trastuzumab resistance in HER2-positive breast cancer. Our findings support rationale for further investigation of NCT-58 as a new therapeutic approach for trastuzumab-resistant HER2-positive breast cancers.

## Materials and methods

### Reagents and antibodies

The synthesis of NCT-58 has been described previously [29]. Primary antibodies targeted Ki-67, CD31, ALDH1 and CD44 (Abcam, MA); HER2, phospho-HER2 (Tyr1221/1222), HER3, phospho-HER3 (Tyr1289), EGFR, phospho-EGFR, Akt, phospho-Akt (Ser473), PARP, cleaved-PARP, cleaved-caspase-3, cleaved-caspase-7, vimentin, Nanog, Oct4, Sox2, Ras, Raf (Ser338), phospho-Raf, Mek, phospho-Mek (Ser217/221), Erk and phospho-Erk (Tyr202/204) (Cell Signaling, CA); CB11 (Thermo Fisher Scientific Fremont, CA); anti-intracellular domain (ICD) HER2 clone 4B5 (Ventana Medical Systems, AZ); survivin, HSP70, HSP90, and HSF-1 (Santa Cruz Biotechnology, CA); and GAPDH (Invitrogen, CA). Secondary antibodies were HRP-conjugated anti-rabbit and mouse IgG (Bio-Rad Laboratories, CA) and Alexa Fluor-488 and −594 goat anti-rabbit IgG (Invitrogen).

### Breast cancer cell culture

The human breast cancer cell lines BT474, SKBR3, MDA-MB-453 (American Type Culture Collection, ATCC), JIMT-1 (DSMZ GmbH, Germany), and MDA-MB-231 (PerkinElmer, Inc., CT) were cultured in DMEM, RPMI 1640 or MEM (Gibco, MD) containing 10% fetal bovine serum (FBS) and penicillin-streptomycin (100 U/mL). The normal human mammary epithelial cell line MCF10A (ATCC) was cultured in Mammary Epithelial Cell Growth Medium (MEGM), including hEGF, insulin, hydrocortisone, and bovine pituitary extract (SingleQuots^TM^ Kit, Lonza, CA) with streptomycin-penicillin (100 U/mL). Cells were incubated at 37 °C in an atmosphere of 5% CO_2_.

### Stable HER2 and p95HER2 overexpression in MDA-MB-231 cells

HER2- and p95HER2-overexpressing MDA-MB-231 cells were generated using a lentiviral system, as previously described [51, 52]. Briefly, the HER2 or p95HER2 gene was amplified by PCR [51, 52] and then inserted into a dual promoter lentivector (CD550A-1, System Biosciences, USA). After transfection, puromycin (final concentration, 3 μg/mL) selection was performed and single colonies were isolated from a dish.

### Cell viability assay

Cell viability was measured using the CellTiter 96* Aqueous One Solution Cell Proliferation Assay [MTS, 3-(4,5-dimethylthiazol-2-yl)-5-(3-carboxymethoxyphenyl)−2-(4-sulfophenyl)−2H-tetrazolium] (Promega, WI), as previously described [53].

### Cell cycle analysis and Annexin V/PI assay

Cells were harvested and fixed with 95% ethanol containing 0.5% Tween-20 for 24 h, and incubated with propidium iodide (PI, 50 mg/mL) and RNase (50 mg/mL) for 30 min. For the Annexin V/PI assay, a FITC-conjugated Annexin V apoptosis detection kit (BD Biosciences, Franklin Lakes, NJ) was used according to the manufacturer’s protocol. Stained cells were analyzed by flow cytometry using BD LSRFortessa^TM^ X-20 Cell Analyzer (BD Biosciences).

### Aldefluor-positivity assay and CD44/CD24 staining

ALDH1 activity was determined using an Aldefluor assay kit (Stemcell Technologies, Vancouver, BC), as previously described [43]. Diethylamino-benzaldehyde (DEAB) was used to define the Aldefluor-positive population. For CD44/CD24 staining, cells were stained with FITC- and PE-conjugated anti-mouse IgG or FITC-conjugated anti-CD24 and PE-conjugated anti-CD44 antibodies (BD Biosciences) and analyzed with a BD LSRFortessa™ X-20 Cell Analyzer.

### HSP90α (C-Terminal) inhibitor screening assay

An HSP90α (C-Terminal) Inhibitor Screening Assay Kit (BPS Bioscience, CA) was used to assess recombinant C-terminal domain of HSP90α:PPID binding activity, as previously described [51]. All assays were performed with an Optiplate-384 (PerkinElmer) and measured using an AlphaScreen® microplate reader (Varioskan LUX™, Thermo Fisher Scientific, Rockford, IL).

### Western blot analysis

The procedures were performed as previously described [54]. Primary antibody dilutions were as follows: HER2 (1:5000), phospho-HER2 (1:1000), HER3 (1:2000), phospho-HER3 (1:2000), EGFR (1:2000), phospho-EGFR (1:2000), Akt (1:2000), phospho-Akt (1:2000), Ras (1:3000), Raf (1:1000), phospho-Raf (1:500), Mek (1:2000), phospho-Mek (1: 1000), Erk (1:2000), phospho-Erk (1:2000), survivin (1:2000), PARP (1:2000), cleaved-PARP (1:2000), cleaved-caspase-3 (1:2000), cleaved-caspase-7 (1:2000), HSF-1 (1:2000), HSP70 (1:3000), HSP90 (1:3000), ALDH1 (1:2000), Nanog (1:2000), Oct4 (1:2000), Sox2 (1:2000) or GAPDH (1:3000), followed by incubated with HRP-conjugated rabbit or mouse secondary antibodies (1:3000–1:10,000). Signal intensity was detected using a Chemiluminescence Kit (Thermo Fisher Scientific) on X-ray film (Agfa Healthcare, Mortsel, Belgium) and quantitated using AlphaEaseFC software (Alpha Innotech, San Leandro, CA).

### Immunocytochemistry

The procedures were performed as previously described [55]. Primary antibodies ALDH1 (1:100), HSP90 (1:300), HSP70 (1:100), HSF-1 (1:100), HER2 (1:100), vimentin (1:200) or ICD-HER2 (1:100) in antibody diluent (Dako, Glostrup, Denmark) were incubated overnight at 4 °C and then reacted with fluorescence-conjugated secondary antibody (Alexa Fluor®-594 or −488). Cells were mounted with ProLong Gold Antifade Reagent with DAPI (Life Technologies, CA). Images were acquired using a Carl Zeiss confocal microscope and fluorescence intensity of HSP90, HSP70, HSF-1 or HER2 was analyzed by confocal microscopy using the intensity profiling tool.

### Cell sorting and cytological centrifugation

BT474 and JIMT-1 cells were incubated for 45 min at 37 °C in Aldefluor assay buffer containing the ALDH protein substrate BODIPY-aminoacetaldehyde (BAAA). Aldefluor-positive (ALDH1+) or -negative (ALDH1−) populations were sorted by FACSMelody cell sorters (BD Bioscience). For immunocytochemistry, the sorted cells (2.4 × 10^4^) were attached to a glass slide by cytospin centrifugation (Hanil Science; Daejeon, Korea), fixed with 4% paraformaldehyde, washed with PBS, and permeabilized with 0.02% Triton X-100 for 10 min. The cells were incubated with the primary antibody [ALDH1 (1:100), HER2 (1:100), HSF-1 (1:100) or HSP70 (1:100)] in antibody diluent at 4 °C overnight and then reacted with Alexa Fluor® 488- or 594-conjugated secondary antibodies at RT for 2 h. Cells were mounted with DAPI, and images were acquired using a Carl Zeiss confocal microscope.

### Mammosphere formation assay in vitro

BT474 (5 × 10^4^/mL) and JIMT-1 (5 × 10^4^/mL) cells were plated in ultralow attachment dishes and cultured in HuMEC basal serum-free medium (Gibco), supplemented with B27 (1:50, Invitrogen), 20 ng/mL basic fibroblast growth factor (bFGF, Sigma), 20 ng/mL human epidermal growth factor (EGF, Sigma), 4 μg /mL heparin, 1% antibiotic-antimycotic, and 15 μg/mL gentamycin at 37 °C in an atmosphere of 5% CO_2_. The number and volume of the mammospheres were determined under an Olympus CKX53 inverted microscope. The volumes of mammospheres were calculated using the formula Volume = 4/3*3.14(π) * r^3^ (r: radius).

### In vivo xenograft and mammosphere formation assays

All animal procedures were conducted in accordance with the *Guide for the Care and Use of Laboratory Animals*, approved by the Korea University Institutional Animal Care and Use Committee (IACUC, KOREA-2018-0135). Five-week-old female BALB/c nude mice were obtained from the Shizuoka Laboratory Animal Center (Shizuoka, Japan) and housed in a specific pathogen-free environment. JIMT-1 cells (3 × 10^6^) were implanted into the right fourth mammary fat pads of 6-week-old BALB/c nude female mice. When average tumor volumes reached 100 mm^3^, the animals were randomized into 2 groups (*n* = 6/each group), solvent control (DMSO/Corn oil, 1:9) or NCT-58 (30 mg/kg BW) was administered intraperitoneally every other day for 47 days. Tumor volumes were measured twice weekly after the initial treatment and calculated using the formula V = (Length × Width^2^)/2.

For the in vivo mammosphere-forming assay, tumors were harvested when volumes reached 200–250 mm^3^ and dissociated with type III collagenase (2 mg/mL). The digested tissues were filtered through a 40 mm cell strainer, centrifuged at 200 × *g* for 5 min and washed with medium containing 0.2% bovine serum albumin (BSA). Dissociated single cells (5 × 10^4^) were plated in ultralow attachment dishes and cultured in the presence or absence of NCT-58 (5 and 10 µM) for 8 days.

### Serum biochemistry profiles for biomarkers of liver and renal injury

At sacrifice, blood samples from each animal were collected, and serum enzyme activities of aspartate aminotransferase (AST), alanine aminotransferase (ALT), and blood urea nitrogen (BUN) levels were determined with an assay kit following the manufacturer’s protocol (Sigma-Aldrich).

### Immunohistochemistry and in situ localization of apoptosis (TUNEL)

Immunohistochemistry analysis was performed as previously described [44]. Tissue sections were incubated with primary antibodies [Ki-67 (1:100), CD31 (1:100), HER2 (1:100), 4B5 (1:100), CD44 (1:100), ALDH1 (1:100), HSF-1 (1:100), HSP70 (1: 100) or HSP90 (1:300)] in antibody-diluent overnight at 4 °C, and then reacted with Alexa Fluor® 488- or 594-conjugated secondary antibodies at RT for 2 h, followed by ProLong gold antifade reagent with DAPI (Life Technologies). In situ TUNEL was carried out on tissue sections using a TUNEL kit (Roche Applied Sciences, Penzberg, GER). All images were taken with a confocal microscope. The fluorescence intensities of HER2, ICD-HER2, CD44, ALDH1, HSF-1, HSP70, or HSP90 were analyzed using a histogram tool in the Carl Zeiss software package.

### Statistical analysis

All data were analyzed using GraphPad Prism 5.0 statistical software (San Diego, CA). The results are presented as mean ± SD of at least three independent experiments. Data were analyzed by student’s *t*-test, and one- or two-way ANOVA as appropriate. Significance between multiple experimental groups was determined using the Bonferroni *post hoc* test and defined at *p* < 0.05.

## Supplementary information

Supplementary Figure Legends

Supplementary Figure S1

Supplementary Figure S2

Supplementary Figure S3

Supplementary Figure S4

Supplementary Figure S5

Supplementary Figure S6

Supplementary Figure S7

Supplementary Figure S8

## Data Availability

All data generated or analyzed during this study are included in this published article.

## References

[1] Schopf FH, Biebl MM, Buchner J (2017). The HSP90 chaperone machinery. Nat Rev Mol Cell Biol..

[2] Wu J, Liu T, Rios Z, Mei Q, Lin X, Cao S (2017). Heat Shock Proteins and Cancer. Trends Pharm Sci..

[3] Trepel J, Mollapour M, Giaccone G, Neckers L (2010). Targeting the dynamic HSP90 complex in cancer. Nat Rev Cancer.

[4] Calderwood SK, Gong J (2016). Heat shock proteins promote cancer: it’s a protection racket. Trends Biochem Sci..

[5] Citri A, Gan J, Mosesson Y, Vereb G, Szollosi J, Yarden Y (2004). Hsp90 restrains ErbB-2/HER2 signalling by limiting heterodimer formation. EMBO Rep..

[6] Citri A, Kochupurakkal BS, Yarden Y (2004). The achilles heel of ErbB-2/HER2: regulation by the Hsp90 chaperone machine and potential for pharmacological intervention. Cell Cycle.

[7] Calderwood SK, Khaleque MA, Sawyer DB, Ciocca DR (2006). Heat shock proteins in cancer: chaperones of tumorigenesis. Trends Biochem Sci..

[8] Lavaud P, Andre F (2014). Strategies to overcome trastuzumab resistance in HER2-overexpressing breast cancers: focus on new data from clinical trials. BMC Med.

[9] Vu T, Claret FX (2012). Trastuzumab: updated mechanisms of action and resistance in breast cancer. Front Oncol..

[10] Arteaga CL, Sliwkowski MX, Osborne CK, Perez EA, Puglisi F, Gianni L (2011). Treatment of HER2-positive breast cancer: current status and future perspectives. Nat Rev Clin Oncol..

[11] Pohlmann PR, Mayer IA, Mernaugh R (2009). Resistance to Trastuzumab in Breast Cancer. Clin Cancer Res.

[12] Parra-Palau JL, Morancho B, Peg V, Escorihuela M, Scaltriti M, Vicario R, et al. Effect of p95HER2/611CTF on the response to trastuzumab and chemotherapy. J Natl Cancer Inst. 2014;106:dju291. 10.1093/jnci/dju291 PMC4271027 25253614

[13] Scaltriti M, Rojo F, Ocana A, Anido J, Guzman M, Cortes J (2007). Expression of p95HER2, a truncated form of the HER2 receptor, and response to anti-HER2 therapies in breast cancer. J Natl Cancer Inst..

[14] Chandarlapaty S, Scaltriti M, Angelini P, Ye Q, Guzman M, Hudis CA (2010). Inhibitors of HSP90 block p95-HER2 signaling in Trastuzumab-resistant tumors and suppress their growth. Oncogene..

[15] Duru N, Candas D, Jiang G, Li JJ (2014). Breast cancer adaptive resistance: HER2 and cancer stem cell repopulation in a heterogeneous tumor society. J Cancer Res Clin Oncol..

[16] Korkaya H, Wicha MS (2013). HER2 and breast cancer stem cells: more than meets the eye. Cancer Res.

[17] Qiu Y, Yang L, Liu H, Luo X. Cancer stem cell-targeted therapeutic approaches for overcoming trastuzumabresistance in HER2-positive breast cancer. Stem Cells. 2021;39:1125–36 10.1002/stem.3381 33837587

[18] Boulbes DR, Chauhan GB, Jin Q, Bartholomeusz C, Esteva FJ (2015). CD44 expression contributes to trastuzumab resistance in HER2-positive breast cancer cells. Breast Cancer Res Treat..

[19] Shah D, Osipo C (2016). Cancer stem cells and HER2 positive breast cancer: the story so far. Genes Dis..

[20] Korkaya H, Paulson A, Iovino F, Wicha MS (2008). HER2 regulates the mammary stem/progenitor cell population driving tumorigenesis and invasion. Oncogene..

[21] Ginestier C, Hur MH, Charafe-Jauffret E, Monville F, Dutcher J, Brown M (2007). ALDH1 is a marker of normal and malignant human mammary stem cells and a predictor of poor clinical outcome. Cell Stem Cell.

[22] Nami B, Wang Z. HER2 in Breast cancer stemness: a negative feedback loop towards trastuzumab resistance. Cancers (Basel). 2017;9:40. 10.3390/cancers9050040 PMC5447950 28445439

[23] Oliveras-Ferraros C, Corominas-Faja B, Cufi S, Vazquez-Martin A, Martin-Castillo B, Iglesias JM (2012). Epithelial-to-mesenchymal transition (EMT) confers primary resistance to trastuzumab (Herceptin). Cell Cycle.

[24] Bradley E, Bieberich E, Mivechi NF, Tangpisuthipongsa D, Wang G (2012). Regulation of embryonic stem cell pluripotency by heat shock protein 90. Stem Cells.

[25] Fernandes CFL, Iglesia RP, Melo-Escobar MI, Prado MB, Lopes MH (2019). Chaperones and beyond as key players in pluripotency maintenance. Front Cell Dev. Biol..

[26] Garcia-Carbonero R, Carnero A, Paz-Ares L (2013). Inhibition of HSP90 molecular chaperones: moving into the clinic. Lancet Oncol..

[27] Wang Y, McAlpine SR (2015). N-terminal and C-terminal modulation of Hsp90 produce dissimilar phenotypes. Chem Commun (Camb.).

[28] Sauvage F, Messaoudi S, Fattal E, Barratt G, Vergnaud-Gauduchon J (2017). Heat shock proteins and cancer: how can nanomedicine be harnessed?. J Control Release.

[29] Nguyen CT, Ann J, Sahu R, Byun WS, Lee S, Nam G (2020). Discovery of novel anti-breast cancer agents derived from deguelin as inhibitors of heat shock protein 90 (HSP90). Bioorg Med Chem Lett..

[30] Sebolt-Leopold JS, Herrera R (2004). Targeting the mitogen-activated protein kinase cascade to treat cancer. Nat Rev Cancer.

[31] Streicher JM (2019). The role of heat shock proteins in regulating receptor signal transduction. Mol Pharm..

[32] Wang B, Lee CW, Witt A, Thakkar A, Ince TA (2015). Heat shock factor 1 induces cancer stem cell phenotype in breast cancer cell lines. Breast Cancer Res Treat..

[33] Magnifico A, Albano L, Campaner S, Delia D, Castiglioni F, Gasparini P (2009). Tumor-initiating cells of HER2-positive carcinoma cell lines express the highest oncoprotein levels and are sensitive to trastuzumab. Clin Cancer Res.

[34] Dontu G, Abdallah WM, Foley JM, Jackson KW, Clarke MF, Kawamura MJ (2003). In vitro propagation and transcriptional profiling of human mammary stem/progenitor cells. Genes Dev..

[35] Charpin C, Devictor B, Bergeret D, Andrac L, Boulat J, Horschowski N (1995). CD31 quantitative immunocytochemical assays in breast carcinomas. Correlation with current prognostic factors. Am J Clin Pathol..

[36] Gomez-Pastor R, Burchfiel ET, Thiele DJ (2018). Regulation of heat shock transcription factors and their roles in physiology and disease. Nat Rev Mol Cell Biol..

[37] Meng L, Gabai VL, Sherman MY (2010). Heat-shock transcription factor HSF1 has a critical role in human epidermal growth factor receptor-2-induced cellular transformation and tumorigenesis. Oncogene..

[38] Xi C, Hu Y, Buckhaults P, Moskophidis D, Mivechi NF (2012). Heat shock factor Hsf1 cooperates with ErbB2 (Her2/Neu) protein to promote mammary tumorigenesis and metastasis. J Biol Chem..

[39] Santagata S, Hu R, Lin NU, Mendillo ML, Collins LC, Hankinson SE (2011). High levels of nuclear heat-shock factor 1 (HSF1) are associated with poor prognosis in breast cancer. Proc Natl Acad Sci USA.

[40] Barna J, Csermely P, Vellai T (2018). Roles of heat shock factor 1 beyond the heat shock response. Cell Mol. Life Sci..

[41] Kim JY, Barua S, Huang MY, Park J, Yenari MA, Lee JE. Heat shock protein 70 (HSP70) induction: chaperonotherapy for neuroprotection after brain injury. Cells. 2020;9:2020. 10.3390/cells9092020 PMC7563654 32887360

[42] Bai X, Ni J, Beretov J, Graham P, Li Y (2018). Cancer stem cell in breast cancer therapeutic resistance. Cancer Treat. Rev..

[43] Kim YJ, Sung D, Oh E, Cho Y, Cho TM, Farrand L (2018). Flubendazole overcomes trastuzumab resistance by targeting cancer stem-like properties and HER2 signaling in HER2-positive breast cancer. Cancer Lett..

[44] Kim JY, Cho Y, Oh E, Lee N, An H, Sung D (2016). Disulfiram targets cancer stem-like properties and the HER2/Akt signaling pathway in HER2-positive breast cancer. Cancer Lett..

[45] Kabakov A, Yakimova A, Matchuk O. Molecular chaperones in cancer stem cells: determinants of stemness and potential targets for antitumor therapy. Cells. 2020;9:892. 10.3390/cells9040892 PMC7226806 32268506

[46] Cho TM, Kim JY, Kim YJ, Sung D, Oh E, Jang S (2019). C-terminal HSP90 inhibitor L80 elicits anti-metastatic effects in triple-negative breast cancer via STAT3 inhibition. Cancer Lett..

[47] Im CN, Yun HH, Lee JH. Heat shock factor 1 depletion sensitizes A172 glioblastoma cells to temozolomide via suppression of cancer stem cell-like properties. Int J Mol Sci. 2017;18:468. 10.3390/ijms18020468 PMC5344000 28241425

[48] Chumsri S, Sperinde J, Liu H, Gligorov J, Spano JP, Antoine M (2018). High p95HER2/HER2 ratio associated with poor outcome in trastuzumab-treated HER2-positive metastatic breast cancer NCCTG N0337 and NCCTG 98-32-52 (Alliance). Clin Cancer Res.

[49] Chou SD, Prince T, Gong J, Calderwood SK (2012). mTOR is essential for the proteotoxic stress response, HSF1 activation and heat shock protein synthesis. PLoS ONE.

[50] Carpenter RL, Paw I, Dewhirst MW, Lo HW (2015). Akt phosphorylates and activates HSF-1 independent of heat shock, leading to Slug overexpression and epithelial-mesenchymal transition (EMT) of HER2-overexpressing breast cancer cells. Oncogene..

[51] Park JM, Kim YJ, Park S, Park M, Farrand L, Nguyen CT (2020). A novel HSP90 inhibitor targeting the C-terminal domain attenuates trastuzumab resistance in HER2-positive breast cancer. Mol. Cancer.

[52] Pedersen K, Angelini PD, Laos S, Bach-Faig A, Cunningham MP, Ferrer-Ramon C (2009). A naturally occurring HER2 carboxy-terminal fragment promotes mammary tumor growth and metastasis. Mol. Cell Biol..

[53] An H, Kim JY, Oh E, Lee N, Cho Y, Seo JH (2015). Salinomycin promotes anoikis and decreases the CD44+/CD24- Stem-Like Population via Inhibition of STAT3 Activation in MDA-MB-231 Cells. PLoS One.

[54] Oh E, Kim JY, Cho Y, An H, Lee N, Jo H (2016). Overexpression of angiotensin II type 1 receptor in breast cancer cells induces epithelial-mesenchymal transition and promotes tumor growth and angiogenesis. Biochim Biophys Acta.

[55] Oh E, Kim JY, Sung D, Cho Y, Lee N, An H (2017). Inhibition of ubiquitin-specific protease 34 (USP34) induces epithelial-mesenchymal transition and promotes stemness in mammary epithelial cells. Cell Signal.

