# Supplementary material for: The C-terminal HSP90 inhibitor NCT-58 kills trastuzumab-resistant breast cancer stem-like cells
Source: Cell Death Discov. 2021 Nov 13;7:354. doi: 10.1038/s41420-021-00743-2 (PMC8590693; doi:10.1038/s41420-021-00743-2)
Supplement: Supplementary file 1 — Supplementary Figure Legends [file 41420_2021_743_MOESM1_ESM.docx]

**Supplementary Figures Legends**

**The C-terminal HSP90 inhibitor NCT-58 kills trastuzumab-resistant breast cancer stem-like cells**

Soeun Park^1,2,6^, Yoon-Jae Kim^1,2,3,6^, Jung Min Park^1,2^, Minsu Park^1,2^, Kee Dal Nam^1,3,^, Lee Farrand^4^, Cong-Truong Nguyen^5^, Minh Thanh La^5^, Jihyae Ann^5^, Jeewoo Lee^5,*^ Ji Young Kim^1,3,*^ and Jae Hong Seo^1,2,3,*^

**Supplementary Fig. S1. Effect of NCT-58 on expression of apoptosis-related proteins in HER2-positive breast cancer cells. A** Immunoblot analyses of expression of cleaved-caspase-3, cleaved-caspase-7, PARP, cleaved-PARP and survivin in BT474 and SKBR3 cells following exposure to NCT-58 (2-10 μM, 72 h). GAPDH was used as an internal loading control. **B** Quantitative graphs represent the ratio of expression of these proteins relative to GAPDH expression in the presence or absence of NCT-58 (**p*<0.05; ***p*<0.01; ****p*<0.001, n=3). The results are presented as mean ± SD of at least three independent experiments analyzed by one-way ANOVA followed by Bonferroni's post hoc test.

**Supplementary Fig. S2. Influence of NCT-58 on expression of HSP70 and HSP90 proteins. A** Immunoblot analyses of expression of HSP70 and HSP90 proteins in BT474, SKBR3 and JIMT-1 cells after exposure to NCT-58 (2-10 μM, 24 h). GAPDH was used as a loading control. **B** Quantitative graphs represent the ratio of HSP70/GAPDH and HSP90/GAPDH expression in the presence or absence of NCT-58 (NS, not significant, versus DMSO control, n=3). The results are presented as mean ± SD of at least three independent experiments analyzed by one-way ANOVA followed by Bonferroni's post hoc test.

**Supplementary Fig. S3. Effect of NCT-58 on expression of apoptosis-related proteins in trastuzumab-resistant JIMT-1 cells. A** Immunoblot analyses of cleaved-caspase-3, cleaved-caspase-7, PARP, cleaved-PARP and survivin in JIMT-1 cells after NCT-58 treatment (2-10 μM, 72 h). GAPDH was used as an internal loading control. **B** Quantitative graphs represent the ratio of expression of these proteins relative to GAPDH expression in the presence or absence of NCT-58 (****p*<0.001, n=3). The results are presented as mean ± SD of at least three independent experiments analyzed by one-way ANOVA followed by Bonferroni's post hoc test.

**Supplementary Fig. S4. NCT-58 downregulates the expression and phosphorylation of HER2, p95HER2, HER3, EGFR and Akt in JIMT-1 cells. A** Immunoblot analyses of expression of HER2, phospho-HER2 (Tyr1221/1222), p95HER2, phospho-p95HER2 (Tyr1221/1222), EGFR, phospho-EGFR (Tyr1068), HER3, phospho-HER3 (Tyr1289), Akt and phospho-Akt (Ser473) in JIMT-1 cells exposed to NCT-58 (2-10 μM, 72 h). GAPDH was used as a loading control. **B** Quantitative graphs represent the ratio of expression of HER2 family members and Akt relative to GAPDH expression in the presence or absence of NCT-58 (**p*<0.05; ***p*<0.01; ****p*<0.001, n=3). The results are presented as mean ± SD of at least three independent experiments and analyzed by one-way ANOVA followed by Bonferroni’s *post hoc* test.

**Supplementary Fig. S5. Influence of NCT-58 on expression of HSP70 and HSP90 proteins in JIMT-1 cells. A** Immunoblot analyses of expression of HSP70 and HSP90 proteins in JIMT-1 cells after exposure to NCT-58 (2-10 μM, 72 h). GAPDH was used as an internal loading control. **B** Quantitative graphs represent the ratio of HSP70/GAPDH and HSP90/GAPDH in the presence or absence of NCT-58 (***p*<0.01, n=3). The results are presented as mean ± SD of at least three independent experiments analyzed by one-way ANOVA followed by Bonferroni's post hoc test.

**Supplementary Fig. S6. Effects of NCT-58 on Ras, Raf, phospho-Raf, Mek, phospho-Mek, Erk and phospho-Erk protein expression in JIMT-1 cells. A** Immunoblot analyses of expression of Ras, Raf, phospho-Raf (Ser338), Mek, phospho-Mek (Ser217/221), Erk and phospho-Erk (Tyr202/204) in JIMT-1 cells after exposure to NCT-58 (2-10 μM, 72 h). GAPDH was used as an internal loading control. **B** Quantitative graphs represent the ratio of expression of these proteins relative to GAPDH expression in the presence or absence of NCT-58 (**p*<0.05; ***p*<0.01; ****p*<0.001, n=3). The results are presented as mean ± SD of at least three independent experiments and analyzed by one-way ANOVA followed by Bonferroni’s *post hoc* test.

**Supplementary Fig. S7. Influence of NCT-58 on expression of p95HER2 and Akt proteins in p95HER2-overexpressing MDA-MB-231 cells. A** Immunoblot analyses of expression of p95HER2, phospho-p95HER2, Akt and phospho-Akt in p95HER2-overexpressing MDA-MB-231 cells exposed to NCT-58 (2-10 μM, 72 h). **B** Quantitative graphs represent the ratio of expression of these proteins relative to GAPDH expression in the presence or absence of NCT-58 (***p*<0.01; ****p*<0.001, n=3). The results are presented as mean ± SD of at least three independent experiments and analyzed by one-way ANOVA followed by Bonferroni’s *post hoc* test.

**Supplementary Fig. S8. BCSC-enriched subpopulations harbor higher expression of HER2 and HSR-related proteins. A-D** ALDH1 low (ALDH1-) and high (ALDH1+) populations were sorted from JIMT-1 cells using FACS. Cells were immunostained for ALDH1 (green, **A**), HER2 (green, **B**), HSF-1 (green, **C**) and HSP70 (red, **D**) with DAPI (blue). Fluorescence intensity was analyzed by confocal microscopy using the intensity profiling tool. The straight line (white dotted line) indicates 100 intensity units (y-axis on the left, a range scale 0–260 unit).
