# Supplementary figures and images for: The C-terminal HSP90 inhibitor NCT-58 kills trastuzumab-resistant breast cancer stem-like cells
Source: Cell Death Discov. 2021 Nov 13;7:354. doi: 10.1038/s41420-021-00743-2 (PMC8590693; doi:10.1038/s41420-021-00743-2)

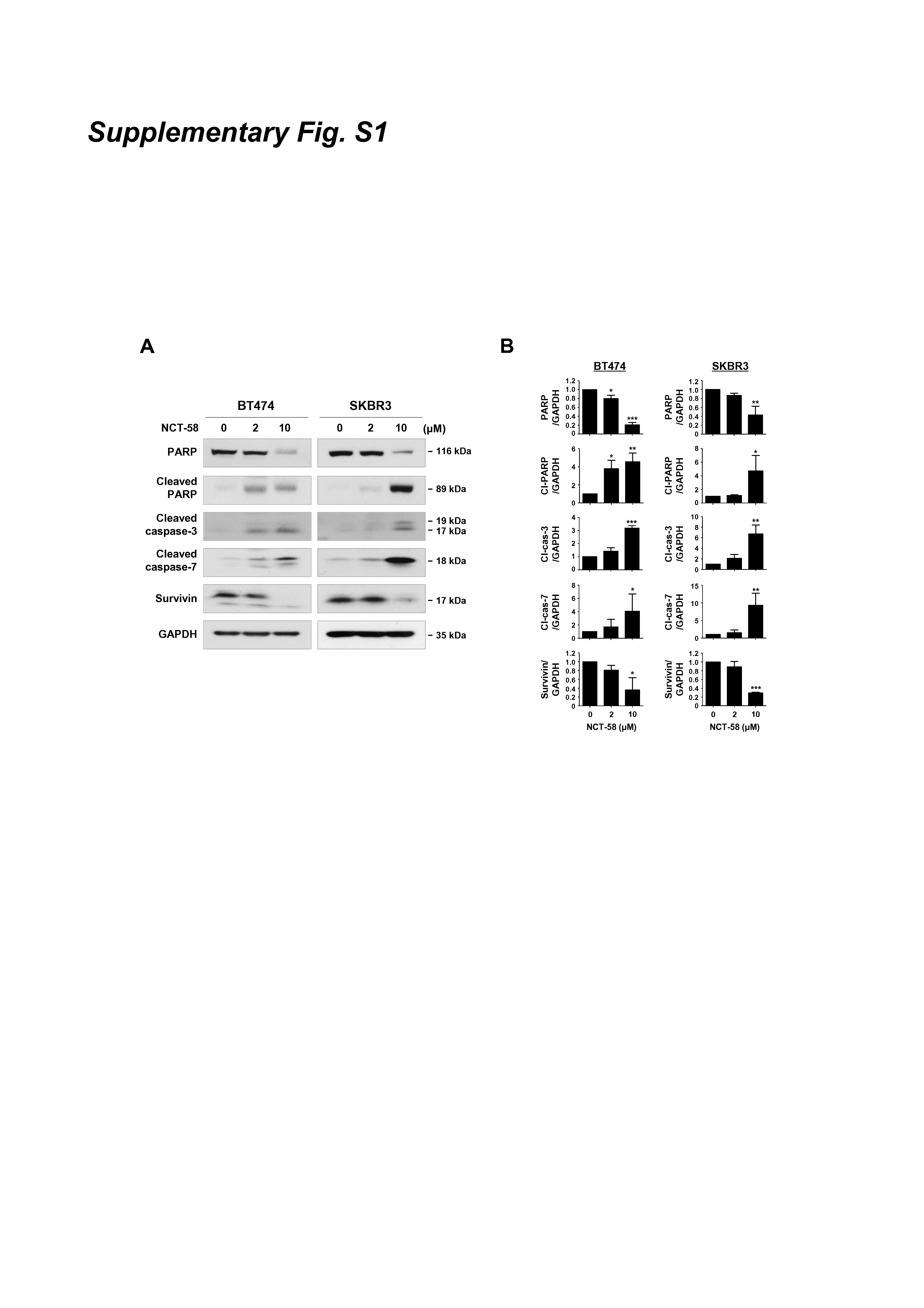

Supplement: Supplementary file 2 — Supplementary Figure S1 [file 41420_2021_743_MOESM2_ESM.tif]

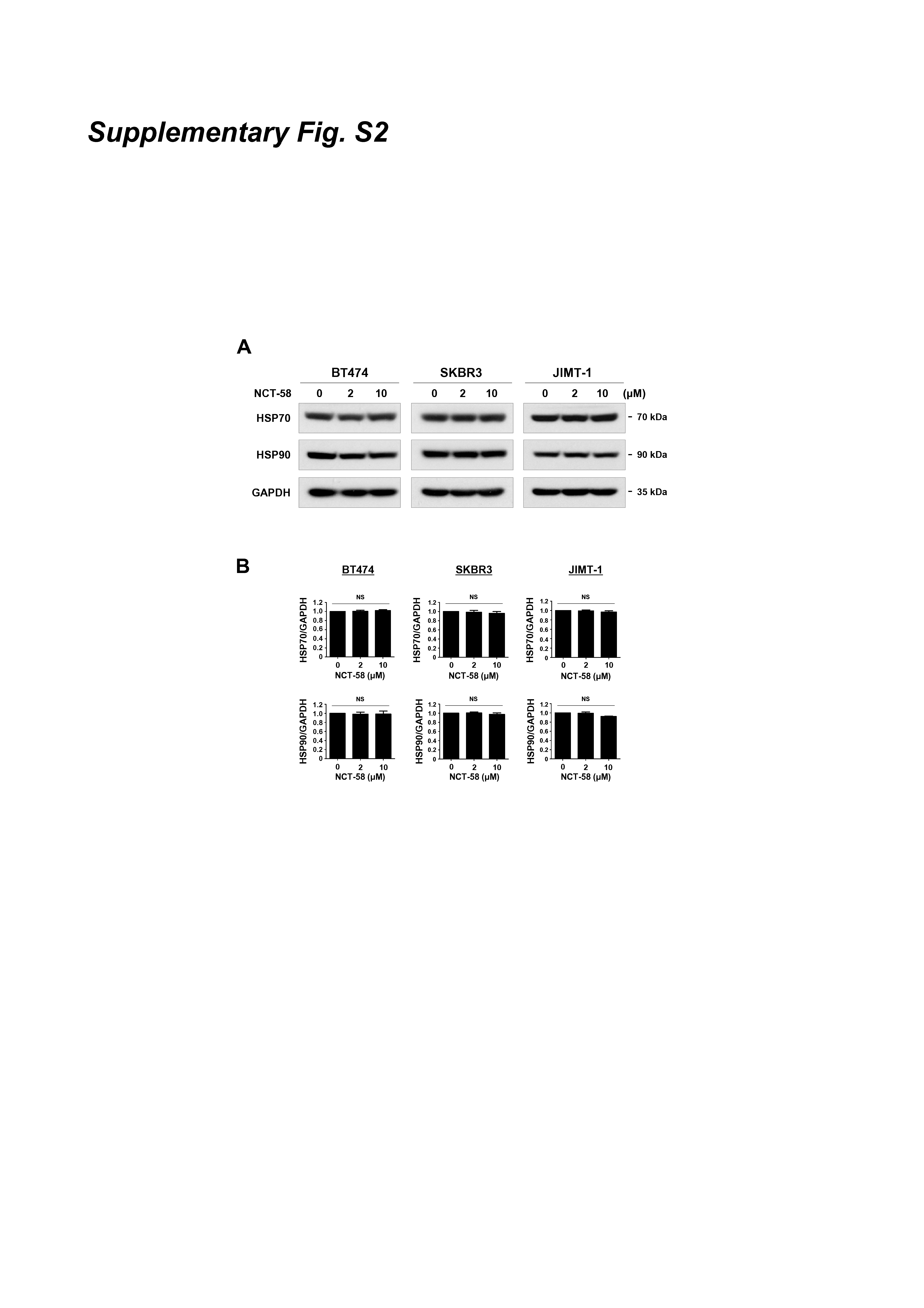

Supplement: Supplementary file 3 — Supplementary Figure S2 [file 41420_2021_743_MOESM3_ESM.tif]

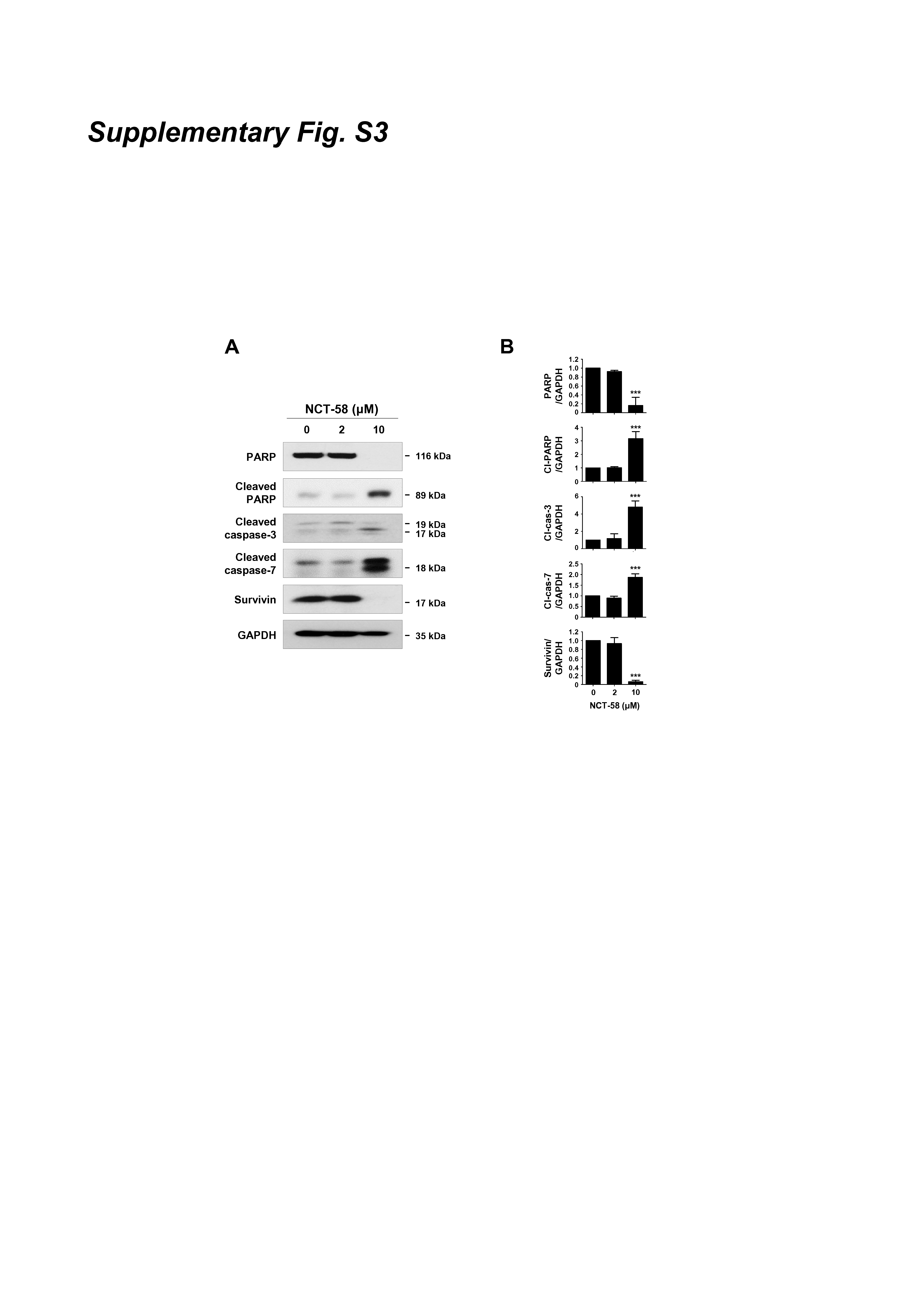

Supplement: Supplementary file 4 — Supplementary Figure S3 [file 41420_2021_743_MOESM4_ESM.tif]

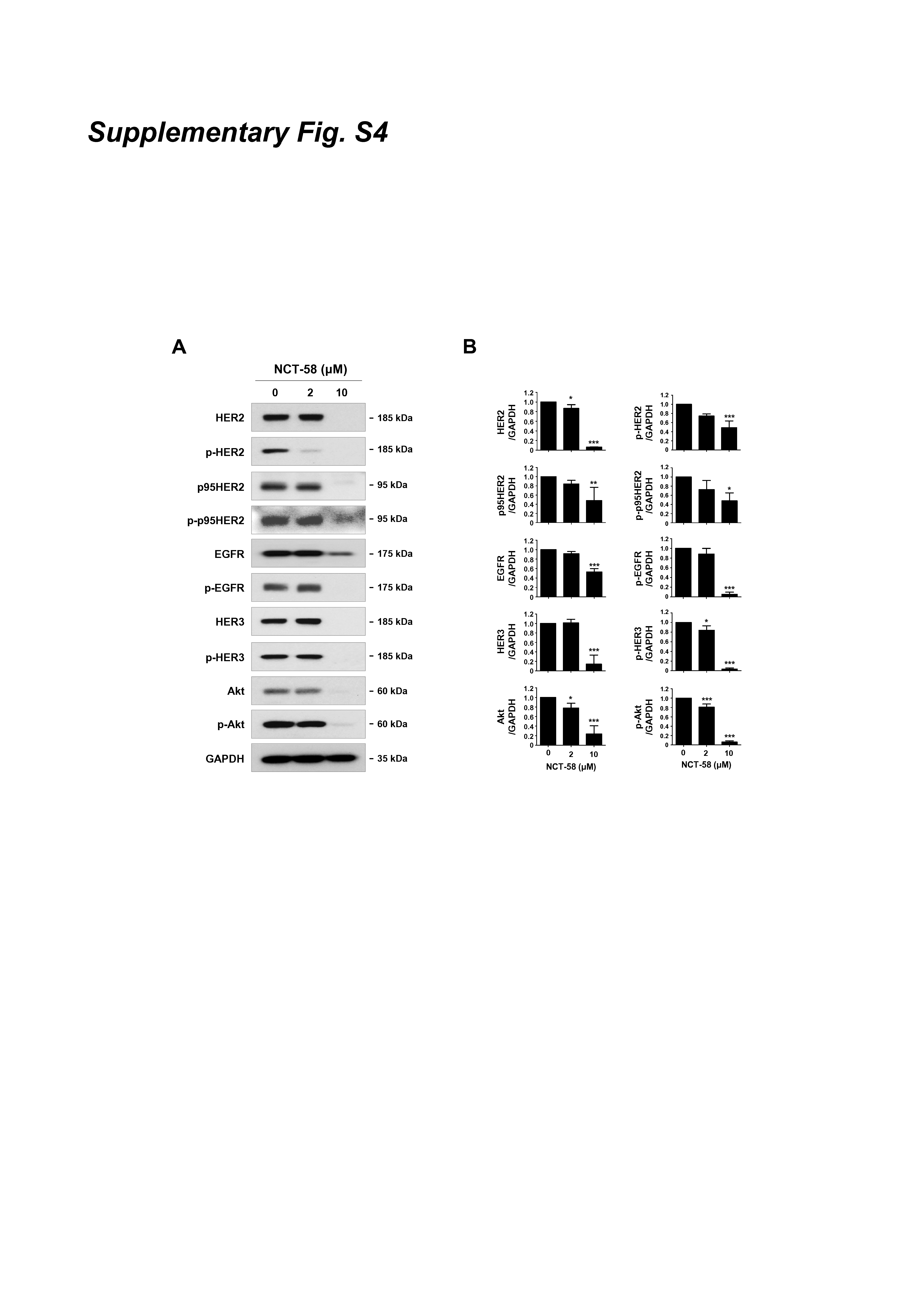

Supplement: Supplementary file 5 — Supplementary Figure S4 [file 41420_2021_743_MOESM5_ESM.tif]

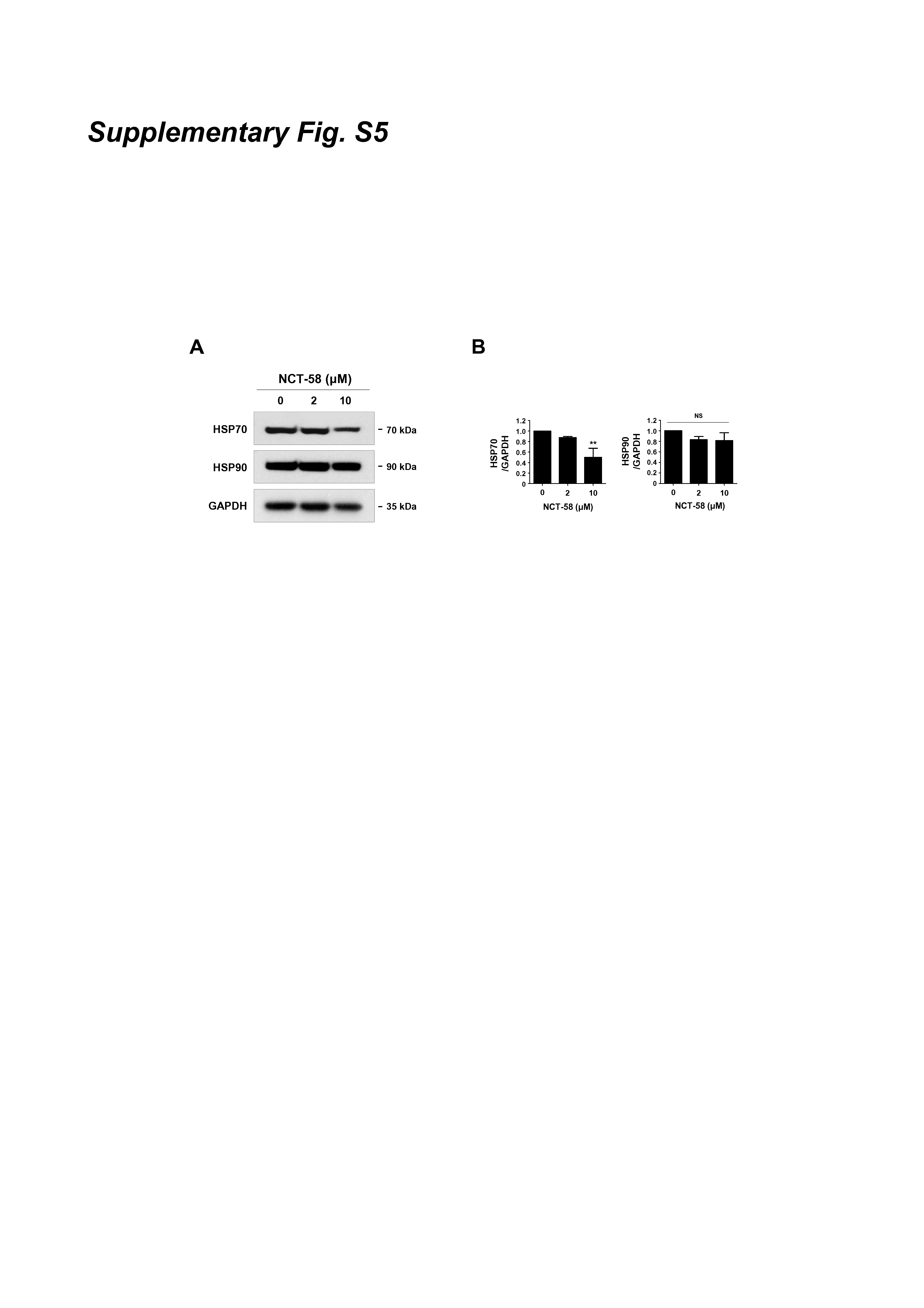

Supplement: Supplementary file 6 — Supplementary Figure S5 [file 41420_2021_743_MOESM6_ESM.tif]

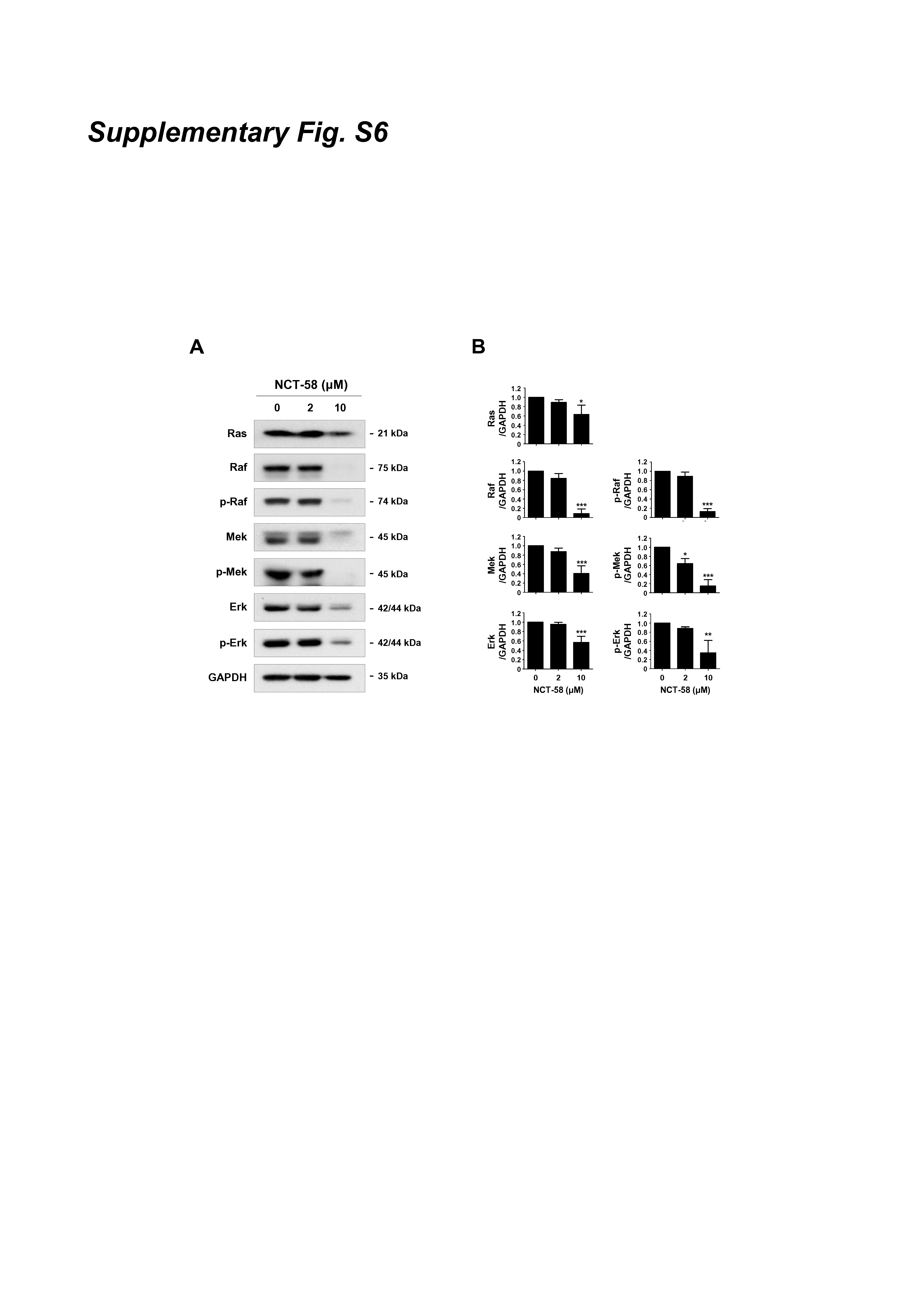

Supplement: Supplementary file 7 — Supplementary Figure S6 [file 41420_2021_743_MOESM7_ESM.tif]

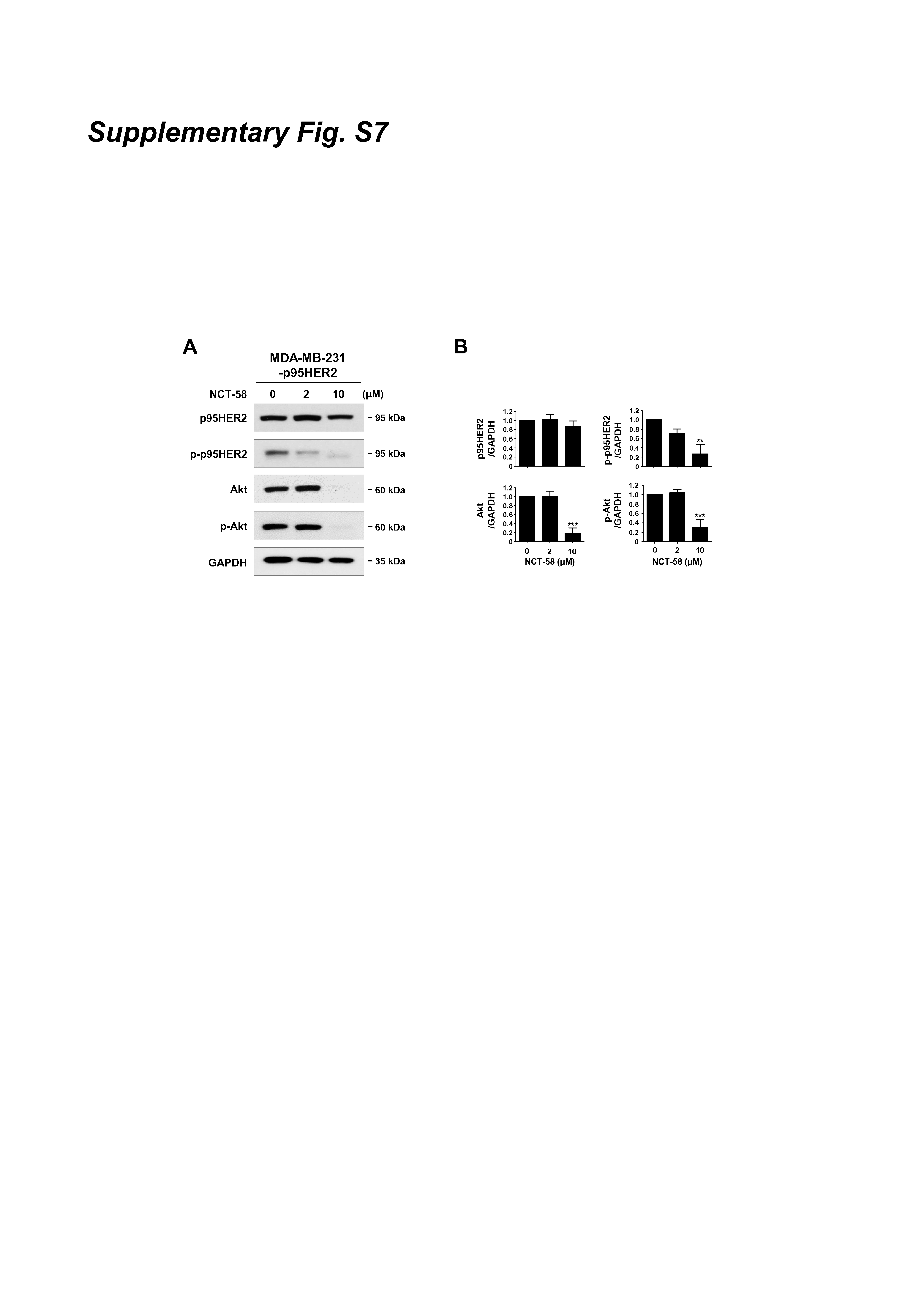

Supplement: Supplementary file 8 — Supplementary Figure S7 [file 41420_2021_743_MOESM8_ESM.tif]

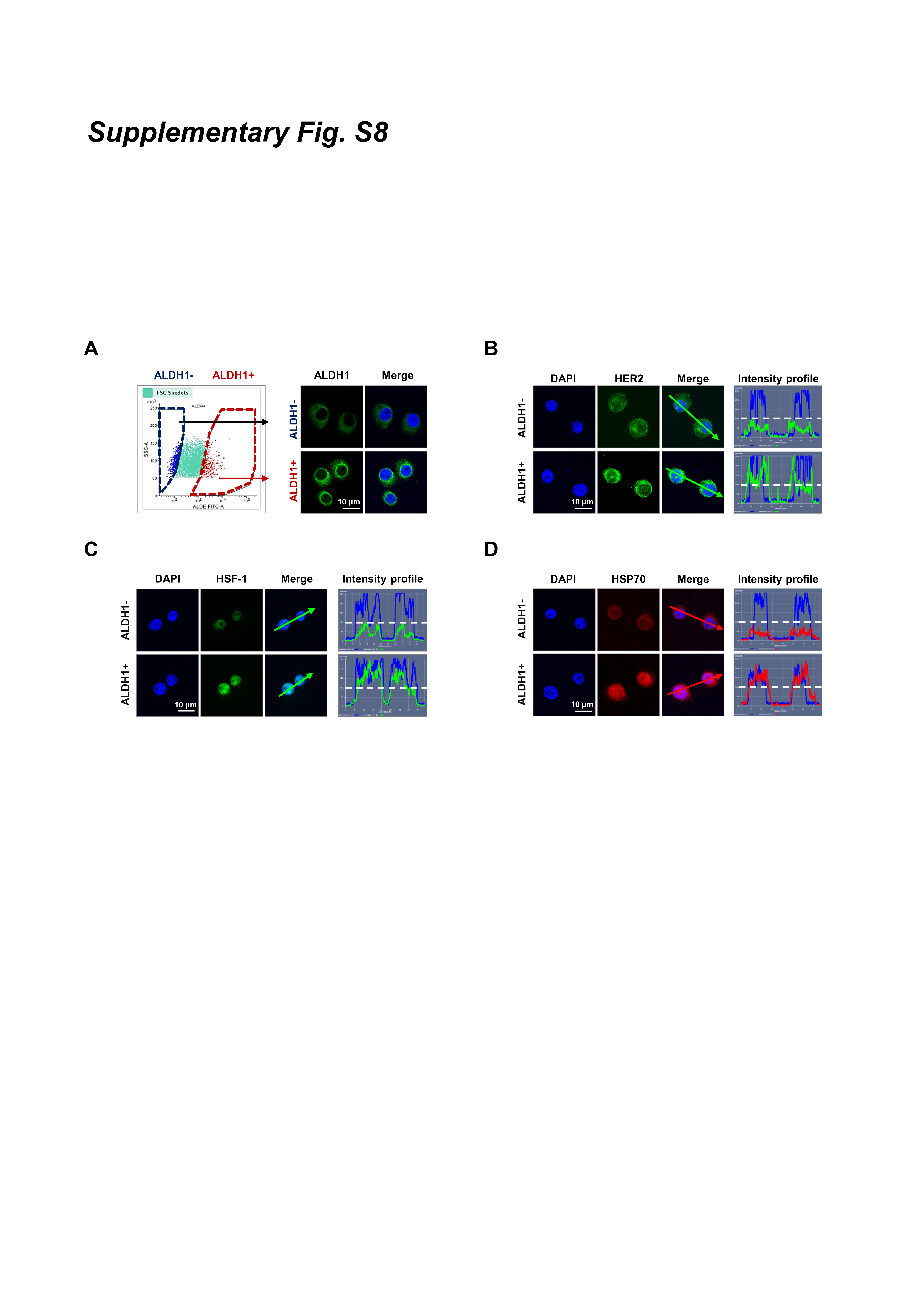

Supplement: Supplementary file 9 — Supplementary Figure S8 [file 41420_2021_743_MOESM9_ESM.tif]
